# Autophagic stress activates distinct compensatory secretory pathways in neurons

**DOI:** 10.1073/pnas.2421886122

**Published:** 2025-07-07

**Authors:** Sierra D. Palumbos, Jacob Popolow, Juliet Goldsmith, Erika L. F. Holzbaur

**Affiliations:** ^a^Department of Physiology, Perelman School of Medicine, University of Pennsylvania, Philadelphia, PA 19104; ^b^Aligning Science Across Parkinson’s Collaborative Research Network, Chevy Chase, MD 20815

**Keywords:** autophagy, secretion, Parkinson’s disease, neurodegeneration

## Abstract

Autophagic dysfunction is a hallmark of neurodegenerative disease, leaving neurons vulnerable to the accumulation of damaged organelles and aggregated proteins. However, the late onset of diseases suggests that compensatory quality control mechanisms may be engaged to delay these deleterious effects. Neurons expressing common familial Parkinson’s disease-associated mutations in the leucine-rich repeat kinase 2 (LRRK2) exhibit defective autophagy. Here, we demonstrate that both primary murine neurons and human induced Pluripotent Stem Cells (iPSC)-derived neurons harboring pathogenic LRRK2 upregulate the secretion of extracellular vesicles. We used unbiased proteomics to characterize the secretome of LRRK2^G2019S^ neurons and found that autophagic cargos including mitochondrial proteins were enriched. Based on these observations, we hypothesize that autophagosomes are rerouted toward secretion when cell-autonomous degradation is compromised to mediate clearance of undegraded cellular waste. Immunoblotting confirmed the release of autophagic cargos and live-cell imaging demonstrated that secretory autophagy is upregulated in LRRK2^G2019S^ neurons. We also found that LRRK2^G2019S^ neurons upregulate the release of exosomes containing microRNAs. Live-cell imaging confirmed that this upregulation of exosomal release is dependent on hyperactive LRRK2 activity, while pharmacological experiments indicate that this release staves off apoptosis. Finally, we show that markers of both vesicle populations are upregulated in plasma from mice expressing pathogenic LRRK2. In sum, we find that neurons expressing pathogenic LRRK2 upregulate secretory autophagy and the compensatory release of exosomes to mediate waste disposal and transcellular communication, respectively. We propose that this increased secretion contributes to the maintenance of cellular homeostasis, delaying neurodegenerative disease progression over the short term while potentially contributing to neuroinflammation over the longer term.

## Significance

A hallmark feature of many neurodegenerative diseases is autophagic dysfunction, resulting in the accumulation of damaged proteins and organelles that is detrimental to neuronal health. The late onset of many neurodegenerative diseases suggests alternative quality control mechanisms may delay neuronal degeneration. Here, we demonstrate that neurons expressing a Parkinson’s Disease-associated mutation upregulate the release of two extracellular vesicle populations. First, we observed the increased expulsion of autophagic vesicles, which mediates cellular waste disposal. Second, we observed the increased release of exosomes, likely to facilitate transcellular communication. Thus, we propose that increases in secretory autophagy and exosome release are homeostatic responses in neurons undergoing chronic stress.

Macroautophagy, hereafter referred to as autophagy, is a fundamental cellular process by which aggregated proteins and dysfunctional organelles are degraded (1). Neurons utilize autophagy as a homeostatic mechanism, exhibiting robust basal autophagy in the absence of cellular stressors. Knockout of critical components of the autophagy machinery, including ATG5 and ATG7, is sufficient to induce neurodegeneration (2, 3). In neurons under basal conditions, cargos to be cleared by autophagy are captured within double membrane autophagosomes that forms preferentially at axonal termini as well as presynaptic sites and are then trafficked back toward the cell soma (4, 5). Autophagosomes fuse with lysosomes en route to the soma in order to degrade internalized cargos (6). Disruption of either autophagosome trafficking or lysosomal fusion inhibits compartment maturation and the subsequent degradation of cargos internalized within axonal autophagosomes (6, 7).

**Fig. 1.**
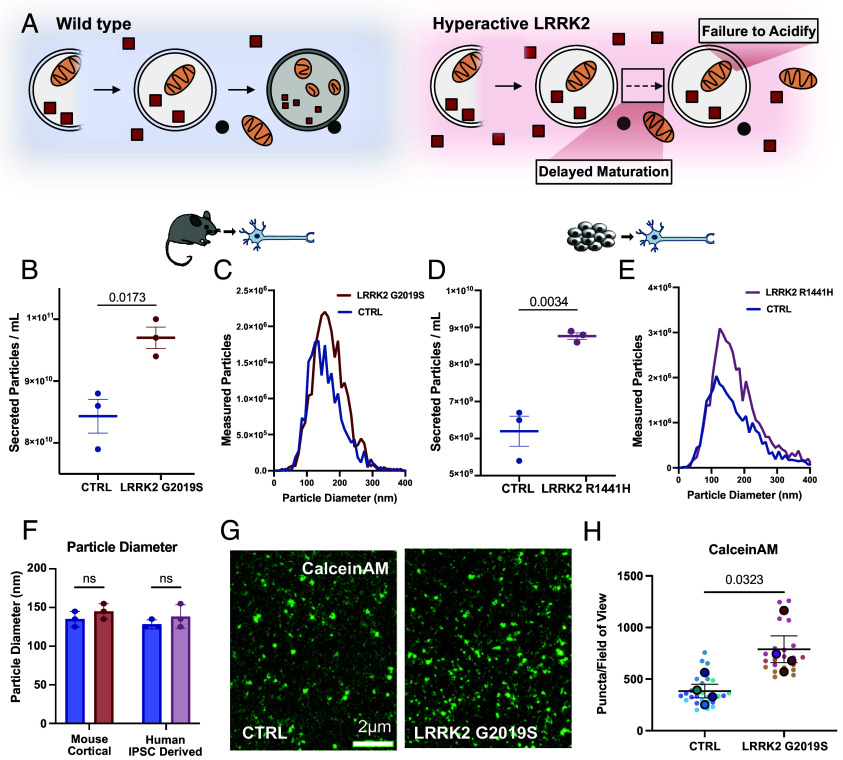
Neurons expressing hyperactive LRRK2 upregulate secretion. (*A*) Degradative autophagy in control and LRRK2 neurons. Neurons expressing hyperactive LRRK2 exhibit delayed autophagosome trafficking and stalled autophagosome maturation. (*B*) Nanoparticle Tracking Analysis of secreted particles isolated from DIV11 wild type and LRRK2^G2019S^ primary cortical neurons. N = 3, two-tailed *t* test, error bars represent mean and SEM. (*C*) Representative size distribution of measured particles released from wild type and LRRK2^G2019S^ primary cortical neurons in individual experiment. (*D*) Quantification of NTA of secreted particles isolated from DIV21 wild type and LRRK2^R1441H^ human KOLF2.1J neurons. N = 3, two-tailed *t* test, error bars represent mean and SEM. (*E*) Representative size distribution of measured particles released from control and LRRK2^R1441H^ human KOLF2.1J neurons. (*F*) Measured particle diameter of the most common particle size (mode) from control and LRRK2 neurons in mouse primary cortical neurons and KOLF2.1J neurons. N = 3, two-way ANOVA with uncorrected Fisher’s least significant difference (LSD), error bars represent mean and SEM. (*G*) Representative TIRF images of vesicles treated with Calcein AM isolated from wild type and LRRK2^G2019S^ cortical neurons. Images pseudocolored. (*H*) Superplot representing measured CalceinAM puncta of vesicles isolated from control and LRRK2^G2019S^ primary cortical neurons. N = 4, two-tailed *t* test comparing biological replicates, error bars represent SEM.

In many neurodegenerative diseases, autophagic dysfunction results in the accumulation of protein aggregates and dysfunctional organelles, threatening neuronal homeostasis (1, 8, 9). Parkinson’s Disease (PD), for example, is a progressive neurodegenerative disease characterized by dopaminergic neuron cell death, autophagic dysfunction, accumulation of *α*-synuclein aggregates, and neuroinflammation (9–11). While many of the phenotypes, e.g., body tremors and bradykinesia, observed in PD can be largely attributed to dopaminergic neuron loss, patients also present with nonmotor symptoms including cognitive impairment and depression, suggesting other neuronal classes are likely impacted (10). Mutations that induce hyperactivity of the kinase LRRK2, including LRRK2^G2019S^ and LRRK2^R1441H^, represent some of the most common causes of familial PD. LRRK2 hyperactivity leads to aberrant phosphorylation of Rab GTPases, key regulators of trafficking pathways in the cell (12, 13). We previously demonstrated that the trafficking and maturation of neuronal autophagosomes were impaired in cortical neurons harboring either the LRRK2^G2019S^ or LRRK2^R1441H^ mutation (Fig. 1*A*) (7, 14–16).

We hypothesized that the resulting strain on autophagy-dependent degradation in neurons might induce activation of alternative quality control mechanisms to prevent or delay proteotoxicity. One possibility is that neurons expressing hyperactive LRRK2 may upregulate secretion, ejecting undegraded cellular waste to the extracellular space via autophagy-dependent secretion. Secretory autophagy is a broad term encompassing multiple pathways that rely on autophagic proteins to mediate the release of diverse contents (17, 18). In canonical secretory autophagy, autophagic proteins mediate the fusion of the outer membrane of an autophagosome with the plasma membrane to release soluble proteins trapped between the vesicular membranes and, presumably, the inner autophagic vesicle (19, 20). Noncanonical secretory autophagy pathways have also been identified that mediate the release of different vesicle or organelle populations, including exosomes (21, 22), exophers (23), and mitochondria (24). Given that inhibition of autophagosome-lysosome fusion has been shown to trigger the upregulation of autophagy-dependent secretion (22, 25–28), we reasoned that neurons expressing pathogenic LRRK2 mutations may similarly engage secretion as a compensatory mechanism in response to autophagic dysfunction.

Here, we demonstrate that neurons expressing hyperactive LRRK2 activate secretory autophagy and the release of exosomes. We performed unbiased proteomic and transcriptomic characterization of vesicles released from LRRK2^G2019S^ neurons, demonstrating that secretory autophagy exports EVs containing the autophagic-membrane-associated protein LC3 and known cargos of autophagy, including synaptic proteins and mitochondria, while we find that the secreted exosomes contain microRNAs (miRNAs) known to regulate autophagy and inflammation. We performed a buoyant linear density gradient and membrane protection assay to confirm that secretory autophagy mediates the **s**ecretion of **L**C3+ **a**utophagic **m**embrane **e**xtracellular **v**esicle**s** (SLAM-EVs) that contain autophagic cargos. We used a knockdown approach to confirm that the release of SLAM-EVs is dependent on the secretory autophagy SNARE protein, SEC22B. We used live-cell imaging to characterize the spatial and temporal dynamics of release of both SLAM-EVs and exosomes. Further, we find that the release of exosomes is neuroprotective. Finally, we show that markers of both vesicle populations are upregulated in plasma from a mouse line expressing pathogenic LRRK2^G2019S^, suggesting an upregulation of secretion is likely observed in vivo. Based on these findings, we propose that activation of these two secretory pathways mediate distinct roles, the release of cellular waste via SLAM-EVs and transcellular communication via exosomes. Together, upregulation of these secretory pathways likely acts as a compensatory mechanism to sustain cellular homeostasis when autophagy-mediated degradation is impaired. Importantly, however, the elevated secretion of proinflammatory contents such as mitochondrial DNA (29, 30) and miRNAs (31, 32) is likely to negatively affect neuronal health over the longer term, contributing to neuroinflammation and disease progression in patients with PD.

## Results

### LRRK2 Mutant Neurons Upregulate Secretion.

Pathogenic mutations in LRRK2 induce kinase hyperactivity, leading to inappropriate phosphorylation of kinase substrates, predominantly organelle-associated Rab GTPases (15), and a resulting disruption in intracellular trafficking pathways (12), including autophagosome maturation in neurons (7, 16). To test our hypothesis that this disruption might lead to a compensatory increase in secretion, we began by focusing on the most common PD-linked mutation in LRRK2, a G2019S missense mutation. We isolated embryonic cortical neurons from wild type and LRRK2^G2019S^ mice. Conditioned media were collected from cultured neurons over 10 d and EVs were enriched from the harvested media. We tracked the size and concentration of particles secreted using Nanoparticle Tracking Analysis (NTA) and observed a 15% increase in particles in EV samples isolated from LRRK2^G2019S^ neurons as compared to EV samples isolated from control neurons (Fig. 1 *B* and *C*). No significant changes in size distribution were detected, with the most commonly detected particle size being 135 nm in wild type and 145 nm in LRRK2^G2019S^ samples (Fig. 1*F*). Several shoulder peaks were also detected in our analysis of both wild type and LRRK2 particles, suggesting the release of multiple vesicle populations over a range of sizes (Fig. 1*C*).

To extend this observation, we examined EV secretion from human iPSC-derived neurons [KOLF2.1J (33)] harboring a distinct PD-causing mutation, LRRK2^R1441H^. This mutation results in a more profound hyperactivation of the LRRK2 kinase as compared to G2019S, leading to more profound deficits in autophagosome trafficking (15, 16). Again, we observed significantly more particles released from LRRK2^R1441H^ neurons as compared to isogenic control neurons (a 42% increase) (Fig. 1*D*), with no change in size distribution detected between the genotypes (Fig. 1*F*). While this is a cross-species comparison, the effect size on secretion appears to correlate with the extent of kinase hyperactivation.

To confirm these findings, we used CalceinAM to assay intact vesicles collected from WT and LRRK2^G2019S^ neurons following EV isolation via ultracentrifugation (34). CalceinAM fluoresces after passively entering an EV that is unpermeabilized and thus the fluorescent signal corresponds to intact, membrane-enclosed structures. Consistent with our NTA results, we observed significantly more CalceinAM puncta in samples collected from LRRK2^G2019S^ mutant neurons (Fig. 1 *G* and *H*), indicating increased secretion of extracellular vesicles. Thus, in both mouse and human neurons, hyperactive LRRK2 induces increased secretion of extracellular vesicles.

### Proteomic Profiling of Offloaded Vesicles.

**Fig. 2.**
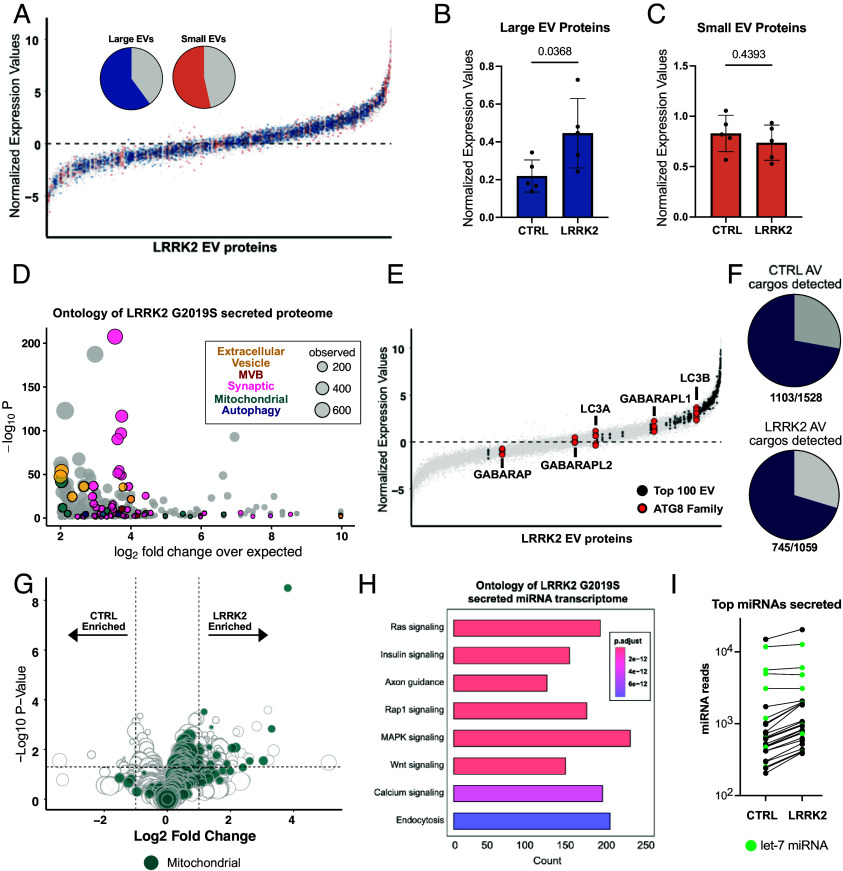
Proteomic profiling reveals autophagic cargo secreted from LRRK2^G2019S^ primary cortical neurons. (*A*) Percent of proteins characterized as “Large EV enriched” (blue) or “Small EV enriched” (orange) by Lischnig et al. (39), detected in LRRK2 secretome. Proteins detected in extracellular vesicles isolated from five replicate samples of LRRK2^G2019S^ neurons ranked by abundance. Median protein abundance = 0. (*B*) Average expression value of all detected Small EV (39) proteins between control and LRRK2 ^G2019S^ secretome. N = 5, two-tailed *t* test, error bars represent SEM. (*C*) Average expression values of all detected Large EV (39) proteins between control and LRRK2 ^G2019S^ secretome. N = 5, two-tailed *t* test, error bars represent SEM. (*D*) Bubble plot representation of ontology terms of top 50% of detected proteins in LRRK2^G2019S^ secretome. Ontology analysis of GO cellular component by PANTHER. Each bubble depicts a unique GO term and the size of bubble represents the number of proteins within the term that was detected. Bubbles pseudo coated by terms. (*E*) Proteins in LRRK2^G2019S^ secretome ranked by abundance. Black dots indicate proteins defined as top 100 EV-associated cargo. Red = ATG 8 family. (*F*) Percent of proteins characterized as control autophagic cargo (41) detected in control EVs (*Top*) and as LRRK2 autophagic cargo detected in LRRK2^G2019S^ EVs (*Bottom*). (*G*) Volcano plot representing differentially secreted proteins from control and LRRK2^G2019S^ neurons. Green indicates mitochondrial associated proteins as defined by MitoCarta 3.0. Size of dot correlates to normalized expression value. (*H*) KEGG analysis of targets of the top miRNAs detected neuronal secretome. The top 25 miRNAs were consistent between both genotypes. (*I*) Average number of reads of top 25 miRNAs detected in control and LRRK2^G2019S^ transcriptome. 4/5 miRNAs are members of the let-7 family.

Extracellular vesicles serve a variety of purposes (35, 36), including waste disposal (23, 37) and transcellular signaling (38), and therefore may contain a wide range of internalized cargos and transmembrane proteins. To characterize the released vesicles, we first performed quantitative proteomic analysis of isolated vesicles from five independent cultures of control and of LRRK2^G2019S^ primary cortical neurons. Extracellular vesicles were enriched via ultracentrifugation to broadly capture released EVs. The protein concentration of isolated EVs was normalized across samples prior to quantitative mass spectrometry analysis (*SI Appendix*, Fig. S1 *A* and *B*). Detected proteins were assigned an expression value based on the number of detected reads within a given sample (median expression value = 0). Vesicle enrichment for both genotypes was confirmed by comparison to previous EV proteomic analysis that characterized proteins found in either small or large vesicle fractions (39) (Fig. 2*A* and *SI Appendix*, Fig. S1*C*). We noted the presence of 89/100 of the most common EV-associated proteins, which were readily detected above the median detection threshold in both control and LRRK2 samples (expression value > 0) (40) (Fig. 2*E* and *SI Appendix*, Fig. S1*D*). Next, we compared the expression of detected proteins associated with either small EVs (Fig. 2*B*) or large EVs (Fig. 2*C*) across genotypes. While we detected no change in the average expression value of small-EV associated proteins, we did detect significantly higher expression values for large-EV associated proteins in LRRK2^G2019S^ samples, suggesting that large EVs were likely enriched in these samples.

Ontology analysis of the secretomes of LRRK2 ^G2019S^ and wild type confirmed that proteins associated with secretion, including those associated with exosome formation, e.g., multivesicular body (MVB) proteins, were readily released (Fig. 2*D* and *SI Appendix*, Fig. S1*E*). Interestingly, we noted that proteins associated with synaptic function (e.g., Synapsin-1), mitochondria (e.g., TOMM40), and autophagy (e.g., GABARAP) were also enriched in the secretomes from both LRRK2 ^G2019S^ and wild type neurons (Fig. 2*D* and *SI Appendix*, Fig. S1*E*). Recently, we used proteomic analysis to define the basal cargos of neuronal autophagosomes and detected both synaptic proteins and mitochondria as enriched autophagosome cargo (41). Given this commonality, we reasoned that these neurons could be releasing a population of autophagic vesicles via secretory autophagy. To test this possibility, we compared all detected proteins in EVs isolated from LRRK2^G2019S^ or control neurons to the previously characterized proteome of autophagosomal cargos from the brain (41). In both cases, over 70% of known autophagic cargos from the brain were detected within our EV proteomic datasets (Fig. 2*F*).

If wild type and LRRK2^G2019S^ neurons are releasing the inner autophagosome vesicle, members of the ATG8 family, well-established markers of autophagosomes, should be detected among the secreted proteins. Consistent with this, nearly all secretory autophagy pathways have reported the release of the autophagic protein LC3B (17, 21, 23, 25, 26), a member of the ATG8 family. In line with previous work, we detected 5 of 6 ATG8s in both the LRRK2^G2019S^ and control samples, with the highest detected being LC3B (Fig. 2*E* and *SI Appendix*, Fig. S1*D*). Thus, unbiased proteomic profiling of the secretomes from either control or LRRK2^G2019S^ primary cortical neurons detected markers associated with multiple classes of extracellular vesicles, including exosomes and secreted autophagic vesicles.

To determine how the secretory proteome was altered in LRRK2^G2019S^ mutants, we performed differential expression analysis and observed 250 upregulated proteins and 80 downregulated proteins in samples from LRRK2^G2019S^ neurons as compared to control neurons (Fig. 2*G*). Ontology analysis of the differentially expressed proteins suggests that mitochondrial proteins are the most prominent cargo upregulated in LRRK2^G2019S^ EVs (*SI Appendix*, Fig. S1*F*). Indeed, 92/250 upregulated secreted proteins are classified as mitochondrial by MitoCarta3.0 (42). This observation could indicate that secretory autophagy is upregulated by LRRK2^G2019S^ neurons as 20% of all neuronal autophagosome cargo is mitochondrially derived (41). The contents of LRRK2^G2019S^ autophagosomes are largely unchanged from wild type, with mitochondria being readily targeted for autophagosome engulfment (43). Further, while autophagosome maturation is impaired in LRRK2^G2019S^ neurons, overall numbers of autophagosomes are not significantly impacted. Thus, our findings suggest that LRRK2^G2019S^ neurons, which exhibit autophagosome maturation defects, may be redirecting unacidified autophagosomes toward extracellular release.

### Transcriptomic Profiling of Released Vesicles.

In addition to proteins, many extracellular vesicles contain RNAs, including miRNAs, to mediate transcellular communication. We therefore performed unbiased small RNA (smRNA) sequencing of isolated vesicles from both LRRK2^G2019S^ and wild type neurons. Secreted extracellular vesicles were isolated via ultracentrifugation followed by RNA extraction, smRNA library preparation, and subsequent sequencing of the normalized libraries. In EVs isolated from both wild type and LRRK2^G2019S^, miRNAs were detected, with 391 and 499 miRNAs reaching our detection threshold (average of at least two reads across five samples), respectively. Kyoto Encyclopedia of Genes and Genomes (KEGG) analysis of the downstream targets of the top 25 miRNAs detected in EVs isolated from both wild type and LRRK2^G2019S^ neurons confirmed that the secreted vesicles were likely mediating transcellular communication (Fig. 2*H*). Many signaling pathways, including those that impact inflammatory responses, e.g., MAPK, were enriched in both the wild type and LRRK2^G2019S^ secretome (44). In both genotypes, 4 of the top 5 detected miRNAs were of the *let-7* miRNA family (Fig. 2*I*), which play a role in many processes, including autophagy and inflammation (45, 46). Thus, both wild type and LRRK2^G2019S^ neurons are offloading miRNAs which could be relevant to neuronal homeostasis. We next asked whether any miRNAs were differentially expressed, and detected no miRNAs significantly upregulated in either genotype (*SI Appendix*, Fig. S2*A*). While no individual miRNA was significantly upregulated in LRRK2 EVs, the average levels of the top 50 detected miRNAs were all elevated in LRRK2 compared to wild type (Fig. 2*I* and *SI Appendix*, Fig. S2*B*). Together, our immunoblots and transcriptomics results argue that it is the overall quantity, and not the contents, of released vesicles that are altered in LRRK2^G2019S^ neurons.

### Hyperactive LRRK2 Enhances the Secretion of Distinct Classes of Extracellular Vesicles.

**Fig. 3.**
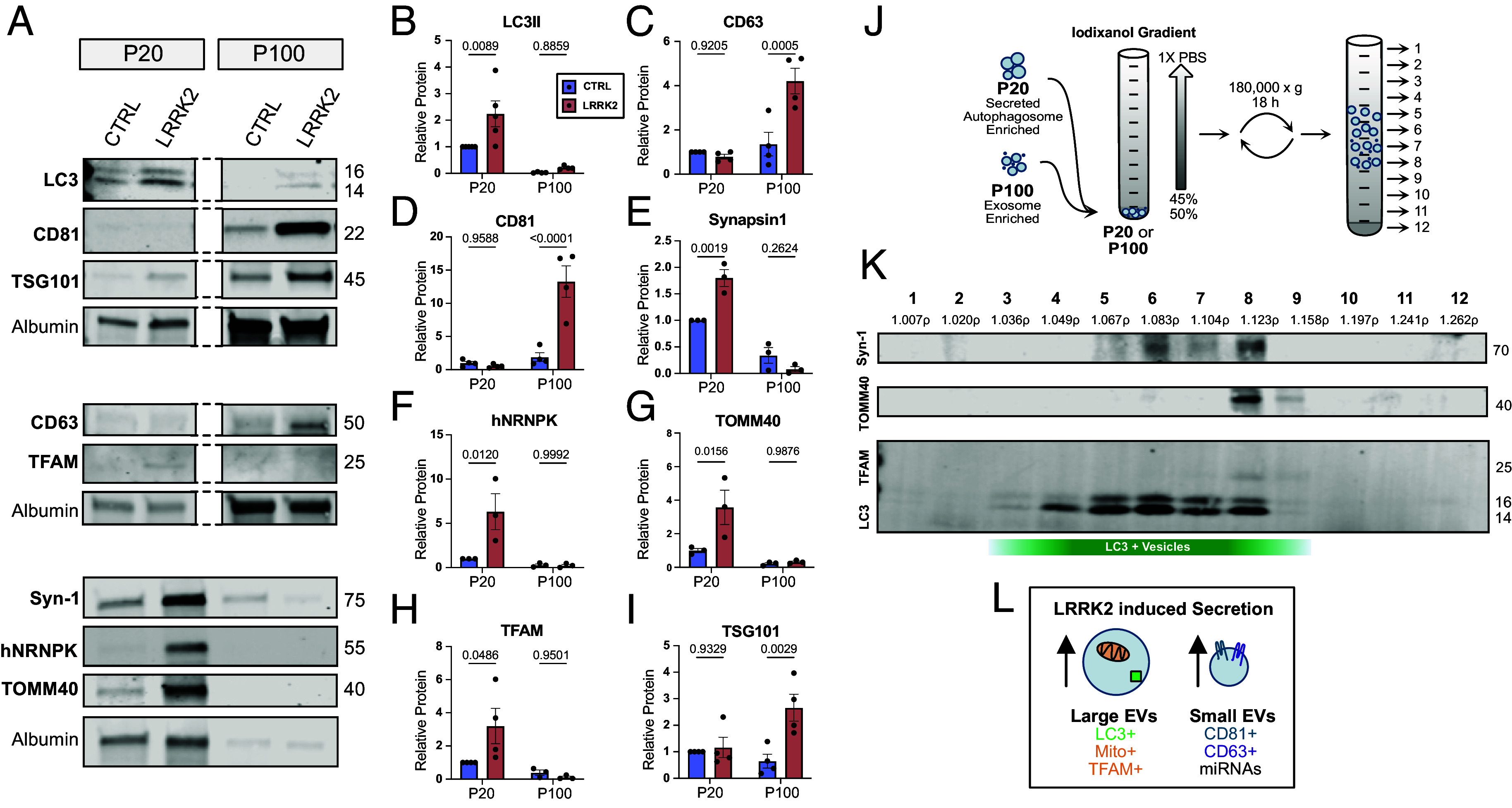
Two secretory pathways are upregulated in LRRK2^G2019S^ neurons. (*A*) Western blots of P20 and P100 fractions from control and LRRK2^G2019S^ neurons. Dashed lines indicate same gel. Equal volume loaded for each well, samples normalized to the amount of detected media protein, albumin. (*B*–*I*) Quantifications of relative levels of detected band intensities for (*B*) LC3-II, (*C*) CD63, (*D*) CD81, (*E*) Synapsin-1, (*F*) hNRNPK, (*G*) TOMM40, (*H*) TFAM and (*I*) TSG101 from P20 and P100 isolated from control (blue) and LRRK2^G2019S^ (red) neurons. All band intensities normalized to the control P20 band to indicate relative abundance. Two-way ANOVA with Šídák’s multiple comparison test. Error bars indicate SEM. (*J*) Buoyant linear density gradient. Isolated vesicle populations (P20 or P100) were bottom loaded in 50% iodixanol below linear gradient (45 to 0%). 180,000× g for 18 h before individual fractions were isolated for immunoblotting analysis. (*K*) Immunoblot of P20 vesicles subfractionated by buoyant linear density gradient. LC3 detected in fractions 3-9, Synapsin-1 fractions 5-8, TOMM40 fractions 8-9, and TFAM fractions 8-9. Measured densities indicated below. (*L*) Secretory autophagy vesicles (*Left*) contain LC3-II, mitochondria, synaptic proteins, and RNA-binding proteins. Exosomes express CD81, CD63, and TSG101.

To identify the class(es) of extracellular vesicles being offloaded from LRRK2^G2019S^ neurons, we performed differential ultracentrifugation of conditioned media to separate EV populations based on density. Conditioned medium was collected and then subjected to a 500× g spin to remove cellular debris and apoptotic bodies (*SI Appendix*, Fig. S3*A*). Large extracellular vesicles, including microvesicles and secreted autophagic vesicles, were enriched via a 20,000× g spin (large EVs/P20), followed by sequential enrichment of small EVs, including exosomes, via a 100,000× g spin (small EVs/P100). Large (P20) and small (P100) EV pellets were isolated and analyzed by immunoblot using antibodies against known EV-associated transmembrane proteins and cargos (Fig. 3*A*). Given that our proteomics results closely mirrored previous proteomic profiling of brain autophagosomes, we first probed for LC3/ATG8 which is offloaded in many secretory autophagy pathways. LC3-I is conjugated to phosphatidylethanolamine to form LC3-II, which marks the forming autophagosome and stays associated throughout autophagosome maturation (47, 48). Across both genotypes, the majority of both LC3-I and LC3-II was detected in the large EV/P20 fraction. Of note, we observed significantly more LC3-II in EVs isolated from LRRK2^G2019S^ neurons as compared to control neurons in the P20 fraction (2.1-fold increase, *P* = 0.0089) (Fig. 3 *A* and *B*).

Most extracellular vesicles are enriched for a family of transmembrane proteins, the tetraspanins, which form microdomains on vesicles important for biogenesis and cargo sorting. We compared the expression of CD81 and CD63, the two most highly enriched tetraspanins on exosomes (49), on EVs isolated from control vs. LRRK2^G2019S^ neurons and observed no detectable CD81 or CD63 in the large EV/P20 fraction. Thus, the large LC3-II+ vesicles being secreted from LRRK2 mutant neurons lack tetraspanins, an observation consistent with their identification as secreted autophagic vesicles. Strikingly, in the P100 fraction enriched for small EVs, we observed significantly more CD81 (8.4-fold increase) and CD63 (2.6-fold increase) in EVs released from LRRK2 mutant neurons as compared to wild type neurons (Fig. 3 *C* and *D*). Changes in the relative enrichment of these proteins were not due to changes in protein expression as no significant changes were observed by immunoblot analysis of neuronal lysates (*SI Appendix*, Fig. S3 *C* and *D*).

Together, these observations suggest that two populations of EVs are upregulated in LRRK2 mutants: 1) larger EVs that are positive for LC3-II but lack canonical tetraspanins and 2) small EVs enriched for CD63 and CD81. Given our proteomic observations suggesting that autophagic cargo are expelled from LRRK2 neurons and previous literature that has found CD63 expression largely restricted to exosomes, we propose that these two EV populations (large EVs and small EVs) represent the SLAM-EVs and exosomes, respectively.

### Large and Small EVs Contain Distinct Cargos.

To provide additional insight into the nature and function of the two populations of secreted vesicles detected, we used immunoblotting to compare cargos associated with either SLAM-EVs or exosomes. Given the size and density of autophagosomes (50), we first asked whether known autophagosome cargos were enriched in our P20 fraction. Previously, our lab defined the contents of neuronal autophagosomes under basal conditions and found that both synaptic proteins, including Synapsin-1, and mitochondria enriched in the nucleoid marker Transcription Factor A, Mitochondrial (TFAM) are targeted to autophagosomes under basal conditions (41). More recently, we found that several cargos are increased in autophagosomes isolated from the brains of mice expressing mutant LRRK2, including the RNA-binding protein, Heterogeneous nuclear ribonucleoprotein K (hnRNPK) (43). hnRNPK has been shown to interact with LC3 and is secreted within EVs released via LC-3- Dependent Extracellular vesicle Loading and Secretion (LDELS) (26). Consistent with our model that hyperactive LRRK2 activity induces the release of secreted autophagic vesicles, we saw significant increases in the release of Synapsin-1 (1.8-fold increase), TOMM40 (3.6-fold increase) TFAM (3.2-fold increase), and hnRNPK (5.9-fold increase) in the LRRK2 P20 pellet as compared to controls when measured by immunoblotting (Fig. 3 *E*–*H*). In contrast, these proteins were minimally detected in the P100 fractions from either LRRK2^G2019S^ or wild type neurons.

Exosomes originate in the MVB, where different contents, e.g., ESCRT proteins and RNAs, are loaded into small vesicles before being released into the extracellular space (51). We tracked the expression of the ESCRT protein and canonical EV cargo, TSG-101, and observed a 3.5-fold increase in the small EV/P100 fraction in LRRK2^G2019S^ mutants (Fig. 3*I*). In contrast, we observed no significant change in enrichment of TSG-101 in the P20 fraction corresponding to large EVs. Exosomes are very small and thus contain limited cargo, often enriched for miRNA and mRNA (51, 52). Our smRNA transcriptomic analysis confirmed that miRNAs were released by both wild type and LRRK2^G2019S^ neurons. To determine whether RNAs were contained within secreted exosomes, we asked whether P100 fractions isolated from wild type or LRRK2^G2019S^ neurons contained RNA. We used the RNA specific dye, SYTO RNASelect (Thermo), to measure relative levels in fluorescence across our different conditions and observed that RNA could be readily detected in both wild type and LRRK2^G2019S^ P100 fractions (*SI Appendix*, Fig. S2*C*), suggesting that RNAs are likely cargos within released exosomes.

Our initial strategy to isolate large EVs/P20 and small EVs/P100 via ultracentrifugation allowed for a crude enrichment of secreted autophagic vesicles and exosomes, respectively, but contain a diverse range of vesicles and secreted organelles. To confirm that the predicted autophagic cargos (Synapsin-1, TOMM40, TFAM) were in the same vesicle population as secreted LC3, we next performed a buoyant linear density gradient to further subfractionate our two vesicle populations. Isolated large EVs/P20 or small EVs/P100 were bottom loaded onto a linear iodixanol gradient followed by an overnight spin at 180,000× g spin to separate vesicles by density (Fig. 3*J*). 12 individual fractions were collected and then concentrated by dialysis before immunoblotting. We observed that LC3 was detected in fractions 3-9 (density range: 1.020ρ to 1.158ρ), confirming that LC3 containing vesicles were buoyant and likely diverse (Fig. 3*K*). We next looked at the predicted autophagosome cargos and found that both Synapsin-1 (density range:1.067ρ to 1.123ρ) and mitochondrial markers, TOMM40 and TFAM (density range: 1.123ρ to 1.158ρ), were detected in overlapping fractions (Fig. 3*K*). Interestingly, TOMM40 and TFAM were detected in a more limited density range, suggesting SLAM-EVs containing mitochondria may be more dense. These results suggest a diverse class of LC3 containing vesicles that range in density, likely due to the enclosed cargos. To confirm we are isolating exosomes, we also performed a buoyant linear density of our P100 fraction and observed the majority of CD63 and CD81 signal at 1.083ρ to 1.123ρ, consistent with previous reports (53) (*SI Appendix*, Fig. S3*B*). Thus, we identified two vesicle populations, secreted autophagic vesicles, or SLAM-EVs, and exosomes, both of which are upregulated in LRRK2^G2019S^ neurons (Fig. 3*L*).

### LRRK2 Mutant Neurons Release SLAM-EVs.

**Fig. 4.**
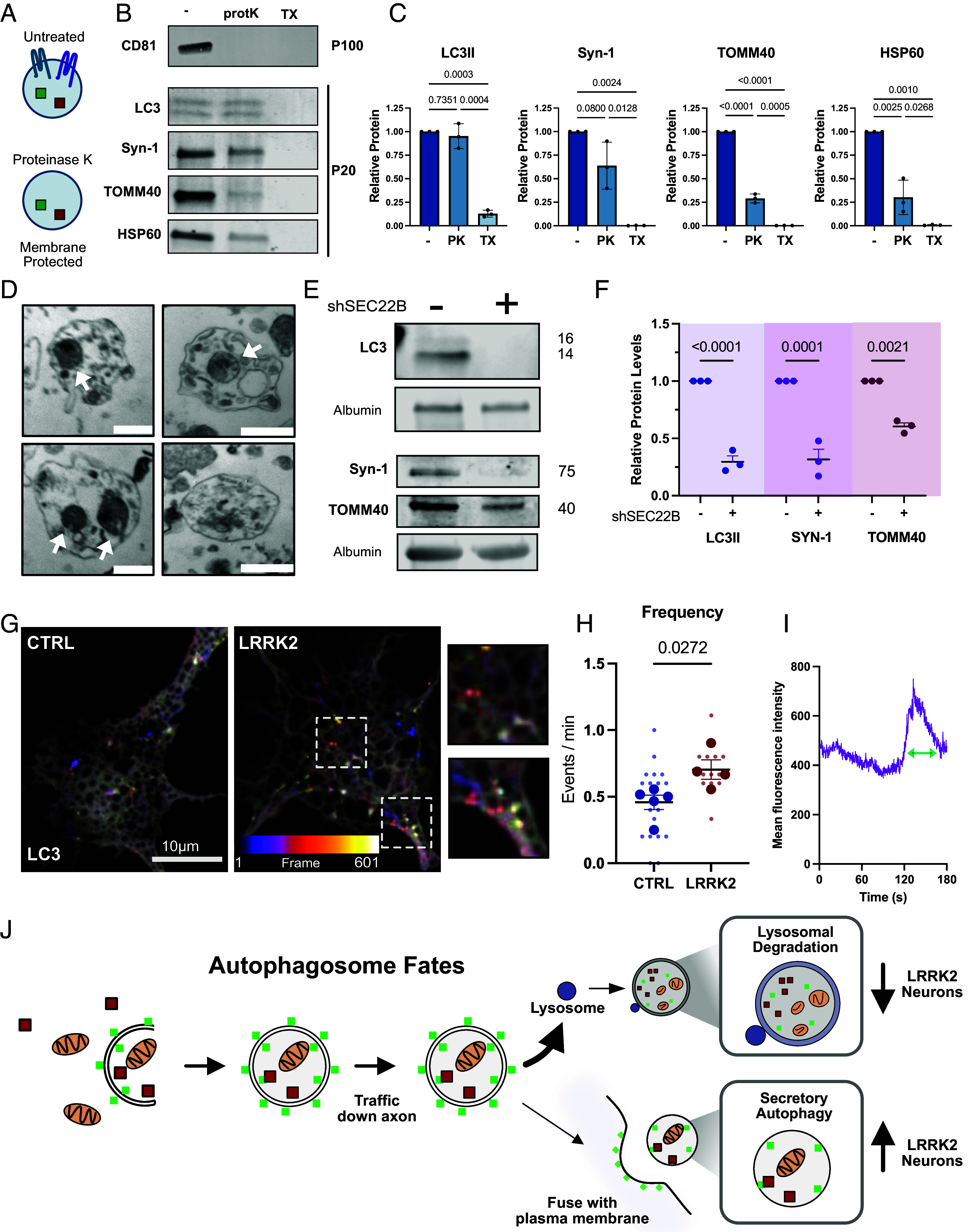
Secretory autophagy is upregulated in LRRK2^G2019S^ neurons. (*A*) Schematic representing outcome following Proteinase K (PK) treatment. (*B*) Immunoblots of isolated vesicles divided into three samples: untreated (−), Proteinase K (protK), or Triton X (TX). Digestion of CD81 used as positive control as antibody against CD81 recognizes epitope on external surface of vesicles. (*C*) Quantification of relative amount of protein in untreated P20 vesicles (−), Proteinase K treated (PK), and Triton X treated. Percent of membrane protected: LC3II (95%), Syn-1 (64%), TOMM40 (30%), and HSP60 (30%). Band intensities normalized to untreated fraction. One-way ANOVA with Türkiye’s multiple comparison test. Error bars indicate SEM. (*D*) P20 isolated vesicles captured with TEM. White arrows indicate dense structures reminiscent of mitochondrial fragments. (Scale bars, 500 nm.) (*E*) Western blots of P20 from LRRK2^G2019S^ neurons treated with control shRNA or *Sec22b* shRNA. Equal volume loaded for each well and samples normalized to the amount of detected media protein, albumin. (*F*) Relative levels of LC3-II, Synapsin-1, and TOMM40, from LRRK2^G2019S^ P20. Two-way ANOVA with Šídák’s multiple comparison test. Error bars indicate SEM. (*G*) Frame sequence of control and LRRK2^G2019S^ neurons transfected with LC3mScarlet. Temporal color code represents frames from 1-601 (2 Hz). Dashed boxes indicate example LC3mScarlet fluorescent event on soma and primary dendrite. (*H*) Quantification of LC3mScarlet fluorescent events per minute in control and LRRK2^G2019S^ neurons. N = 4, two-tailed *t* test comparing biological replicates, error bars represent SEM. (*I*) Example trace of fluorescence intensity of individual LC3mScarlet puncta measured over time. (*J*) Schematic representing the fates of an autophagosome.

While the release of IL-1β, which resides between the interluminal space of the double membrane autophagosome, has been characterized (19), the fate of the inner vesicle upon this release is largely speculative. We propose that this inner vesicle is released extracellularly as a single-membrane vesicle we have termed a SLAM-EV. Consistent with this model, a recent study found that neuronal LC3 can transfer to nearby astrocytes in the absence of cell–cell contact, suggesting a secretory transfer mechanism (54). We therefore wanted to confirm that both the autophagy marker LC3 and known cargos of autophagy, e.g., the mitochondrial protein TOMM40, were indeed contained within these extracellular vesicles. We treated the large EVs/P20 fraction with Proteinase K (ProtK) to determine which proteins were membrane protected (Fig. 4*A*). Nearly all LC3II (95%) was still detectable following ProtK treatment, suggesting this marker is membrane-protected within an intact vesicle, consistent with a topology for SLAM-EVs in which this marker is enriched in the inner leaflet of a single-membrane vesicle (20) (Fig. 4 *B* and *C*). We next probed for known autophagic cargos (e.g., Synapsin-1, mitochondrial proteins). We observed a significant fraction of Synapsin-1 that was membrane protected (64%), consistent with the majority of this protein being vesicle enclosed within SLAM-EVs (Fig. 4 *B* and *C*). Interestingly, using markers for both outer (TOMM40) and inner mitochondrial proteins (HSP60), we observed a smaller protected fraction (30%), suggesting that there is a population of secreted mitochondria that are not vesicle protected and thus not ProtK-resistant (55) in addition to a membrane-protected subfraction. To further characterize SLAM-EVs, we performed transmission electron microscopy on the large EVs/P20 fraction. We identified single membrane vesicles of similar size to autophagosomes (~1 to 1.5 micron diameter). Within these vesicles, we could identify round, electron- dense structures reminiscent of mitochondrial fragments observed within autophagosomes isolated from the mouse brain (Fig. 4*D*) (41). Together, our results suggest a subset of released mitochondria are located within SLAM-EVs, representing a mechanism of mitochondrial release.

The R-SNARE protein, SEC22B, associates with autophagosomes to mediate fusion with the plasma membrane and is required for the extracellular release of IL-1β (50, 56). We therefore asked whether SEC22B was required for the release of SLAM-EVs in LRRK2^G2019S^ neurons. We used shRNA to knockdown *Sec22B* expression (65% knockdown) and then collected large EVs/P20. Upon *Sec22B* knockdown, we observed a significant loss of both LC3II (71% reduction) and Syn-1 (68% reduction), consistent with SEC22B being required for secretory autophagy (Fig. 4 *E* and *F*). Interestingly, we observed a significant, but smaller loss of secreted TOMM40 (39% reduction) upon *Sec22B* knockdown (Fig. 4 *E* and *F*). This finding complements our previous result that a subset of secreted mitochondria is contained within SLAM-EVs, while other mitochondria or mitochondrial fragments are secreted via a SEC22B-independent pathway.

ATG8 family members (LC3/GABARAP) are stably associated with both the inner and outer membrane of mature autophagosomes. In canonical secretory autophagy, the outer LC3/GABARAP membrane fuses with the plasma membrane to release both lumen contents and the inner autophagosome vesicle (57). If autophagosome content is secreted in this manner from the cortical neurons examined here, LC3 should be detected at the cell surface upon vesicle fusion. We transfected wildtype and LRRK2^G2019S^ neurons with fluorescently labeled LC3 and tracked expression at the cell surface using live TIRF microscopy. Consistent with secretory autophagy events, we observed frequent LC3 bursts at the cell surface in both control and LRRK2^G2019S^ neurons (Fig. 4*G*). We quantitated presumed secretory autophagy events by identifying stationary bursts of fluorescence that decayed over 10 to 120 s, similar to other SNARE mediated temporal dynamics (58) (Fig. 4*I*). We detected significantly more LC3 release events in LRRK2^G2019S^ neurons (Fig. 4*H* and Movies S1 and S2), with no change in the duration of events (*SI Appendix*, Fig. S4*B*). Interestingly, we noted that LC3 secretion events were spatially enriched within a somatodendritic compartment which often extended into a single tapered process, consistent with the primary dendrite (Fig. 4*G* and *SI Appendix*, Fig. S4*A*). Morphological characterization confirmed that in ~70% of neurons imaged, LC3 secretion events could be observed at the presumed primary dendrite. We transfected neurons with the AIS marker, Ankyrin G (AnkG), and confirmed that the LC3 release events were excluded from the axon (*SI Appendix*, Fig. S4*A*). Thus, LC3 secretion events are spatially restricted to the somatodendritic compartment. This observation mirrors previous observations from live cell imaging indicating that once axonal autophagosomes enter the soma, they are constrained to the somal and dendritic regions (5). Here, we find that in LRRK2^G2019S^ neurons there is an upregulation in the release of inner autophagic vesicles as SLAM-EVs that contain diverse contents, including synaptic proteins and mitochondria, at these sites (Fig. 4*J*).

### LRRK2 Mutant Neurons Upregulate Release of Exosomes.

**Fig. 5.**
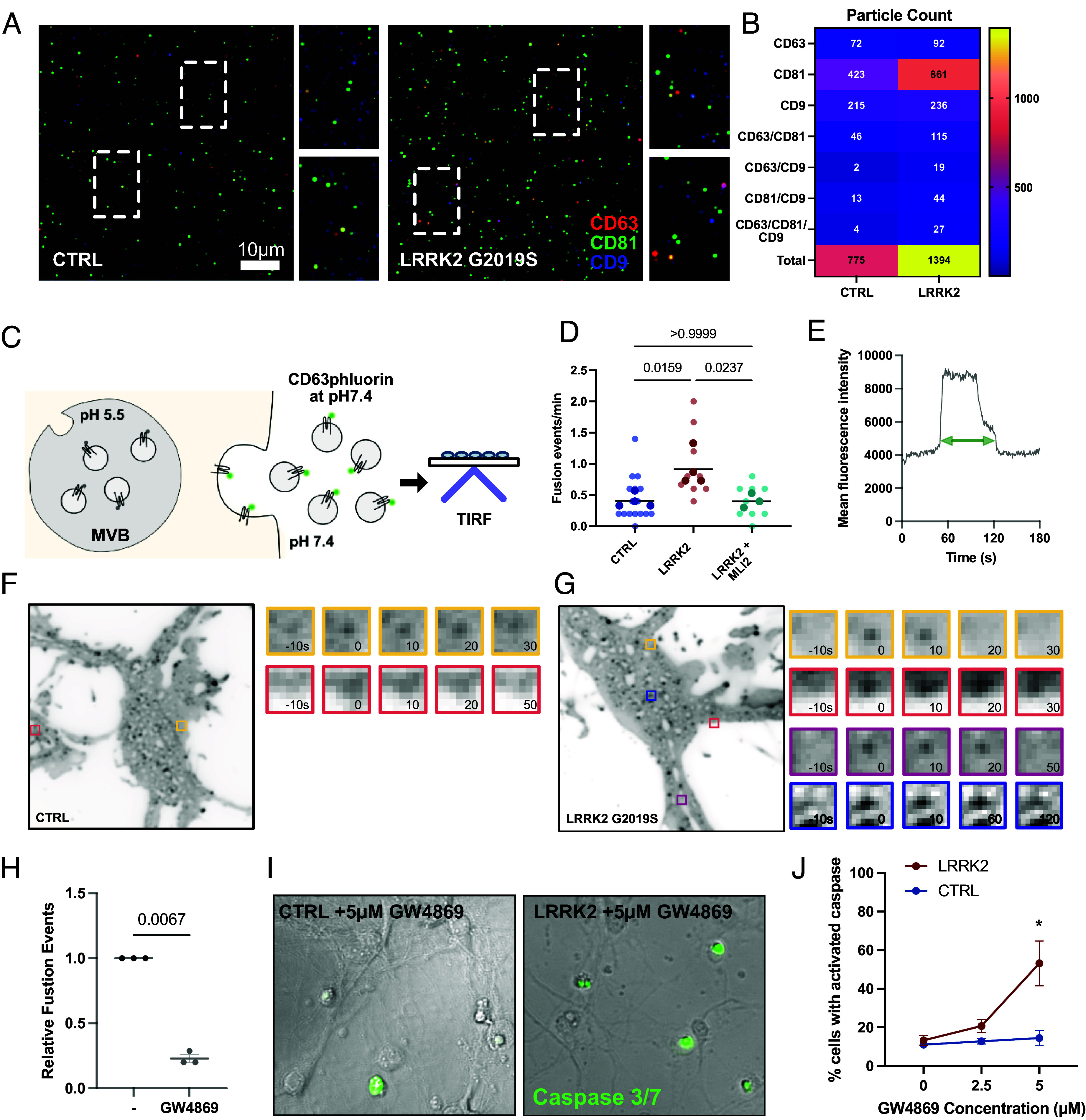
Secretion of exosomes is augmented in LRRK2^G2019S^ neurons. (*A*) Representative image of ExoView anti-CD81 captured vesicles from control and LRRK2^G2019S^ neurons. (*B*) Quantification of detected particles positive for individual or combination of tetraspanins, CD81, CD63, and CD9. (*C*) Pipeline to detect CD63pHluorin secretion events. CD63pHluorin signal is quenched in acidic environments. (*D*) CD63pHluorin events in control, LRRK2^G2019S^, and LRRK2^G2019S^ + MLI-2 primary cortical neurons. N = 4, ordinary one-way ANOVA with Tukey’s multiple comparison test of biological replicates, error bars represent SEM. (*E*) Example trace of fluorescence intensity of individual CD63pHluorin puncta measured over time. (*F* and *G*) Representative image of CD63pHluorin expressing (*F*) control and (*G*) LRRK2^G2019^ neuron. Colored boxes indicate individual secretion events. (*H*) Quantification of CD63pHluorin fusion events before and after application of 5 µM GS4869. N = 3, two-tailed *t* test comparing biological replicates, error bars represent SEM. (*I*) Control and LRRK2^G2019S^ neurons treated with 5 µM of the exosome inhibitor, GW4869. DIC images with overlay of activated CellEvent Caspase3/7. (*J*) Quantification of percent of neurons with activated CellEvent following 2 h of treatment with GW4869. N = 3, two-way ANOVA with Šídák’s multiple comparison test. Error bars indicate SEM.

Our immunoblotting results suggest that small EVs containing both CD81 and CD63 are also secreted from LRRK2^G2019S^ neurons. As immunoblotting relies on the pooling of EVs to look for global changes, we next used ExoView (Spectradyne) to track tetraspanin expression at the level of individual EVs. We captured EVs from both wild type and LRRK2^G2019S^ neurons using an anti-CD81 antibody followed by subsequent antibody detection against CD81, CD9, and CD63 (Fig. 5*A*). Consistent with our earlier observations, more CD81+ EVs were released from LRRK2^G2019S^ neurons compared to wild type neurons (Fig. 5*B*). Additionally, we detected more CD81+/CD63+, and CD81+/CD9+ dual-labeled vesicles captured from LRRK2^G2019S^ EVs (Fig. 5*B*). These observations corroborated our immunoblotting results, where we observed substantial CD81 and CD63 release from LRRK2^G2019S^ neurons, consistent with the release of exosomes.

CD63 is a commonly used marker of exosomes (51, 59). Given our immunoblot results that CD63 is significantly enriched in the small EV/P100 fraction from LRRK2^G2019S^ neurons, we sought to better characterize the secretion of CD63 positive EVs using live cell imaging. We expressed the pH sensitive fluorophore, pHluorin, tagged to CD63 (CD63-pHluorin) in primary cortical neurons isolated from wild type or LRRK2^G2019S^ mice. The modified GFP signal of CD63pHluorin is quenched in acidic environments such as the MVB, and is visible upon fusion with the extracellular membrane for EV release (Fig. 5*C*). We measured CD63-pHluorin signal in control and LRRK2^G2019S^ neurons using TIRF microscopy. New fusion events could be identified by tracking sudden bursts of fluorescence that persisted 10 s to 150 s (Fig. 5 *D*–*G* and *SI Appendix*, Fig. S4*C*). We observed a 2.2-fold increase in fusion events in LRRK2^G2019S^ neurons compared to wild type neurons suggesting that exosomal secretion is upregulated in LRRK2^G2019S^ neurons (Fig. 5*D* and Movies S3 and S4). Additionally, this upregulation of CD63 secretion events is dependent on LRRK2 hyperactivity as pretreatment of neurons with MLI-2, a selective LRRK2 kinase inhibitor (60), blocked the observed increase of CD63 fusion events (Fig. 5*D*). In contrast to the LC3 secretion events described above, CD63-positive release events were primarily observed on the cell soma with no clear spatial clustering near or at the primary dendrite, although occasionally fusion events could be observed on neurites. We confirmed that these events represented exosomes as application of the exosome inhibitor, GW4869, significantly reduced CD63-pHluorin events (Fig. 5*H*). GW4869 blocks the release of exosomes via inhibition of Neutral Sphingomyelinase, a critical component for the budding of intraluminal vesicles into the lumen of the MVB (61).

In addition to the bursts of CD63-pHluorin signal that rapidly decayed, we also noted bursts in fluorescence that persisted for minutes, suggesting that CD63 could also be tethered to or integrated into the plasma membrane upon MVB fusion as well as released (*SI Appendix*, Fig. S5 *A*–*E*). We tracked these longer events and observed a fourfold increase in fusion events in LRRK2^G2019S^ neurons compared to wild type neurons; this difference was rescued by pretreatment of the neurons with MLI-2 (*SI Appendix*, Fig. S5*F*), indicating this phenotype was due to hyperactive LRRK2 activity. To determine whether human neurons expressing the LRRK2^R1441H^ mutation also upregulate the release of CD63+ exosomes, we performed live cell imaging of LRRK2^R1441H^ and isogenic control iPSC-derived neurons expressing CD63-pHluorin. Again, we observed significantly more CD63-pHluorin events in LRRK2^R1441H^ neurons compared to wild type neurons (*SI Appendix*, Fig. S5*G*). Thus, hyperactive LRRK2 promotes the secretion of CD63+ EVs in both mouse and human neurons.

### LRRK2 Hyperactivity Prompts the Release of Exosomes via Secretory Autophagy.

While the majority of secreted LC3 was detected in our P20 fraction, we also could detect a significant increase in secreted LC3-II in the LRRK2^G2019S^ P100 fraction in a direct comparison (*P* = 0.0286). Several groups have demonstrated that LC3/ATG8 lipidation and the LC3-conjugation machinery are critical for several secretory autophagy pathways, including LDELS (26), secretory autophagy during lysosomal inhibition (SALI) (25), and apilimod-mediated secretion of exosomes (21). To determine whether the LRRK2-mediated secretion of exosomes was similarly dependent on LC3 conjugation, we knocked down *Atg7* using siRNA (*SI Appendix*, Fig. S5*H*) and tracked exosome release using CD63-pHluorin in LRRK2^G2019S^ neurons. We determined that ATG7 activity was required for LRRK2 mediated exosome release as we observed a 60% reduction in the number of CD-63-pHluorin secretion events in neurons expressing the *Atg7* siRNA (*SI Appendix*, Fig. S5*I*), suggesting that LC3 conjugation was required for the increased exosomal release induced by hyperactive LRRK2.

### Exosomal Release Is Beneficial to LRRK2 Mutant Neurons.

Does the release of exosomes impact neuronal survival? Recently, it has been observed that augmenting exosomal secretion can extend lifespan in several ALS models. To determine whether the observed exosomal release was beneficial to LRRK2^G2019S^ expressing neurons, we treated DIV7 primary neurons with increasing doses of the exosome inhibitor GW4869. Following 2 h of GW4869 exposure, we tracked levels of activated Caspase 3/7. In wild type neurons, short-term, low doses of GW4869 failed to increase the percent of cells with activated Caspase 3/7, suggesting these cells were largely insensitive to the inhibition of exosomal secretion (Fig. 5 *I* and *J*). In contrast, LRRK2^G2019S^ neurons exposed to 5 μM GW4869 exhibited a dramatic increase in Caspase 3/7 activation (13% at baseline vs. 53% at 5 μM) (Fig. 5 *I* and *J*). This observation suggests that LRRK2^G2019S^ neurons rely on exosomal secretion for survival and are thus more sensitive to exosomal inhibition. In contrast, blocking the release of microvesicles using the drug Y27632, a competitive inhibitor of Rho-associated protein kinases ROCK1 and ROCK2, which play a key role in actin filament organization during microvesicle shedding (61, 62). We found that Y27632 treatment had no impact on cell survival in control or LRRK2^G2019S^ neurons (*SI Appendix*, Fig. S6 *A* and *B*). Thus, we find that LRRK2 hyperactive activity promotes the compensatory release of exosomes in neurons, and this release promotes neuronal survival under stress.

### Chronic and Acute Strain on Autophagy Maturation Differentially Influence Secretion.

In LRRK2^G2019S^ neurons, autophagic degradation is chronically strained (7, 16). Previous reports suggest that an acute prevention of autophagosome degradation via Bafilomycin A 1 (BafA1) (63, 64) can also initiate the upregulation of secretion in several different cell types (65, 66). To determine whether hyperactive LRRK2 was promoting secretion of EVs via a similar mechanism as BafA1, we tracked secretion in neurons treated for 2 h with increasing concentrations of BafA1 (0 nM, 50 nM, 500 nM). We observed significantly more particles secreted by BafA1 treated neurons as measured via NTA (*SI Appendix*, Fig. S6 *A* and *B*). We next isolated Large EVs/P20 and Small EVs/P100 released from DMSO and BafA1 treated neurons for immunoblot analysis (*SI Appendix*, Fig. S6*C*). We observed that BafA1 treatment significantly increased the release of CD81 (2×-fold increase) in small EVs (*SI Appendix*, Fig. S6*D*), but, in contrast to LRRK2^G2019S^ neurons, CD63 was not elevated following BafA1 (*SI Appendix*, Fig. S6*E*). BafA1 treated neurons did release more TSG101 selectively within large EVs (*SI Appendix*, Fig. S6*F*). In contrast to LRRK2^G2019S^, BafA1 treatment resulted in no change in LC3II levels in any fraction (*SI Appendix*, Fig. S6*G*), suggesting BafA1 does not prompt the release of SLAM-EVs. These results reveal distinct differences between BafA1-mediated secretion and the enhanced secretion induced by mutant LRRK2 expression in neurons.

As we observed no change in CD63 levels and only moderately increased CD81 signal, we asked whether BafA1 treatment promoted the secretion of the tetraspanin, CD9. We tracked CD9-pHluorin fusion events in primary cortical neurons and observed significantly more fusion events in neurons following BafA1 incubation (*SI Appendix*, Fig. S6 *H* and *I*). In contrast, LRRK2^G2019S^ neurons exhibited no change in CD9-pHluorin events compared to wild type (*SI Appendix*, Fig. S6*J*). Thus, while we observe that hyperactive LRRK2 activity initiates the release of two EV classes, exosomes and SLAM-EVs, BafA1 mediates the release of a distinct EV population.

### Upregulation of Secretion in Hyperactive LRRK2 Mutant Animals.

Our studies on neurons in vitro identified two secretory pathways that are upregulated in both primary mouse cortical neurons and human iPSC-derived glutamatergic neurons expressing PD-associated mutations that induce hyperactive LRRK2 activity. To determine whether each of these pathways were also upregulated in vivo, we compared levels of relevant vesicle markers in plasma isolated from 1 y old LRRK2^G2019S^ and wild type mice.

**Fig. 6.**
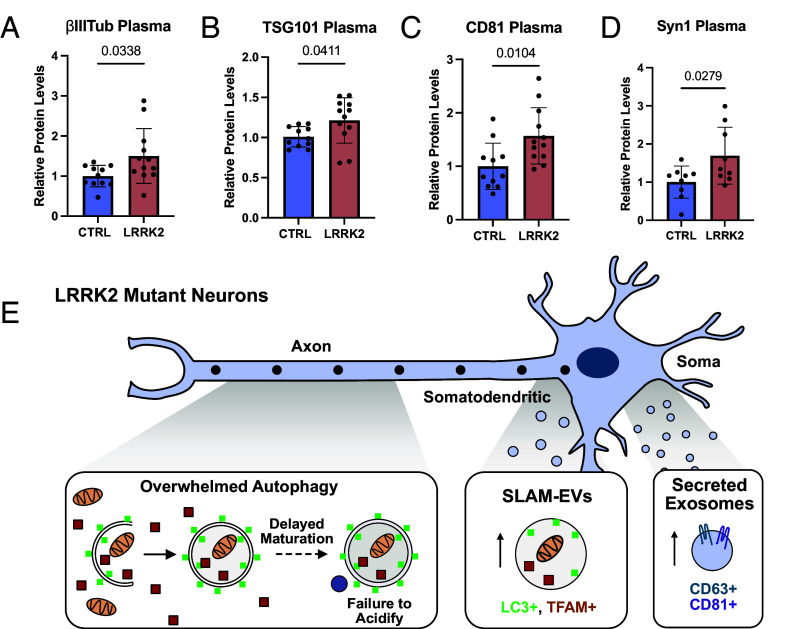
Compensatory release of exosomes and SLAM-EVs. (*A*–*D*) Quantification of band intensity of plasma from control and LRRK2^G2019S^ mice for (*A*) Class III β-tubulin, (*B*) TSG101, (*C*) CD81, and (*D*) Synapsin-1. N = 11, two-tailed *t* test, error bars represent SEM. (*E*) Schematic of proposed model. Neurons expressing pathogenic LRRK2 exhibit delays in axonal autophagosome trafficking and maturation. Two compensatory secretion pathways are upregulated in response to this chronic autophagic stress. SLAM-EVs are preferentially released from a somatodendritic compartment to mediate release of autophagosome cargo. Exosomes are released from the cell soma to likely mediate transcellular signaling.

First, we asked whether overall secretion from neurons was elevated by measuring circulating levels of neuronal protein, BIII-tubulin (*SI Appendix*, Fig. S7 *C*). We detected significantly more BIII-tubulin in LRRK2^G2019S^ plasma compared to wild type plasma (Fig. 6*A*), demonstrating increased neuronal secretion in LRRK2^G2019S^ neurons in vivo. Consistent with a prior report, we also observed more TSG101 in plasma from LRRK2^G2019S^ mice, evidence that overall secretion is elevated by hyperactive LRRK2 activity (Fig. 6*B*). In primary cultured neurons, both CD81 and CD63 were secreted by LRRK2^G2019S^ neurons. In isolated plasma, we observed a significant increase in CD81 in LRRK2^G2019S^ plasma (Fig. 6*C*), but failed to detect any change in CD63 (*SI Appendix*, Fig. S7 *C* and *D*). Together, these observations are consistent with neuronal upregulation of secretion and an upregulation of exosomal release in LRRK2^G2019S^ animals. We next asked whether upregulation of SLAM-EVs could be detected in LRRK2^G2019S^ animals. We tracked circulating levels of the neuronal autophagy cargo Synapsin-1 and found significantly more circulating Synapsin-1 in LRRK2^G2019S^ plasma (Fig. 6*D*). Thus, it is likely that both the exosomal pathway and secretory autophagy pathway are upregulated in LRRK2^G2019S^ animals.

## Discussion

Here, we identify two upregulated secretory pathways, the release of SLAM-EVs and exosomes, that work in tandem to mediate export of cellular waste and intercellular communication, respectively, in PD-neurons facing autophagic stress (Fig. 6*E*); we propose that the increased secretion is a compensatory response to the inhibition of autophagy observed in this model (7, 16). We used both unbiased proteomic and transcriptomic analyses to characterize the cargos of these two vesicle classes, e.g., mitochondria in SLAM-EVs and miRNAs in secreted exosomes. We further corroborated this model using live imaging, demonstrating that the two vesicle classes are both molecularly and spatially distinct. Using a specific LRRK2 inhibitor, we demonstrated that exosomal release is dependent on LRRK2 activity. Finally, we found that neuronal secretion is augmented in animals harboring the LRRK2^G2019S^ mutation and that the secretion of exosomes is critical for LRRK2^G2019S^ neuronal survival in vitro. Together, we define two secretory pathways that are upregulated in neurons expressing mutant LRRK2 and thus undergoing prolonged strain to autophagy-mediated degradation.

We find that autophagosome contents are secreted from neurons harboring hyperactive LRRK2, suggesting that autophagosomes can fuse with the plasma membrane to expel membrane-enclosed inner vesicles and their contents. While this process has been speculated, this work characterizes these vesicles and suggests an alternate fate for autophagosomes that fail to degrade—fusion with the plasma membrane to mediate the release of SLAM-EVs. What happens to the released cellular waste following neuronal expulsion? One possibility is that surrounding glia take up neuronal SLAM-EVs for transcellular degradation. This model is supported by recent findings that neuronal LC3+ vesicles are internalized by neighboring astrocytes (54). Importantly, this study also found that transcellular degradation of neuronal LC3+ vesicles can occur in vivo. A parallel process has been observed for cardiomyocytes, which release large vesicular structures termed exophers for transcellular degradation by nearby macrophages (23). Effective internalization of neuronally secreted mitochondrial fragments by surrounding glia could preclude the activation of harmful neuroinflammatory responses induced by release of mitochondrialDNA (29).

LRRK2^G2019S^ expression upregulates the extracellular release of mitochondria, a population of which resides within SLAM-EVs. Mitochondrial exchange between neighboring cells, including neurons and adjacent glia, has been described by multiple groups (23, 24, 37, 67, 68). Tunneling nanotubes can mediate mitochondrial transfer to rescue neurons facing oxidative stress (69). Neuronal mitochondria can also be transferred to nearby glia for transcellular degradation, though the mechanism is unknown (68). Our findings suggest an additional mode of transfer of mitochondrial fragments from neurons to glia via secretory autophagy. Given that LRRK2^G2019S^ neurons exhibit impaired autophagy (7, 16), neighboring cells may take up and ultimately degrade neuronal cellular waste, including mitochondria, via SLAM-EVs. However, we find that a large portion of secreted mitochondria are not membrane protected, indicating that additional mitochondria secretion pathways, such as autophagic secretion of mitochondria (55), may also play important roles in LRRK2^G2019S^ neurons.

In parallel to the expulsion of SLAM-EVs, we observed a significant increase in exosome secretion. These released exosomes contain RNA, and specifically miRNAs, suggesting that they could be mediating intercellular communication (70). We found this exosomal release to be neuroprotective in monoculture, thus, exosomes could be impacting neighboring neurons. Of interest, we noted that released exosomes are enriched for members of the *let-7* miRNA family. *Let-7* miRNAs are implicated in many processes that impact homeostasis, including the induction of neuronal autophagy (46). Release of *let-7* can be mediated by inflammation (71) and circulating levels of certain *let-7* miRNAs have been found to be elevated in PD patient cerebral spinal fluid (72, 73) and serum (74), suggesting secretion of this family of miRNAs could play a role in disease progression (75). Further work is needed to define the downstream fate and role of both the SLAM-EVs and exosomes in vivo in disease models.

Both genetic (22) and pharmacologic (27, 65, 66, 76, 77) inhibition of autophagosome maturation can trigger the release of vesicles via various secretory autophagy pathways across diverse cell types, thus highlighting the tight interplay between autophagy-dependent degradation and autophagy-dependent secretion. SALI for example, results in the autophagy-dependent-secretion of autophagic cargo receptors in a population of small extracellular vesicles and particles (65). While both SALI and the release of SLAM-EVs redirect autophagic contents toward secretion, SALI relies on the intermediate fusion of the autophagosome with the late endosome, while our data indicate that SLAM-EVs are the result of SEC22B-mediated autophagosome fusion with the plasma membrane. The limited overlap between SALI and SLAM-EV proteomic datasets and the differences in the nature and size of the vesicles further suggest these are distinct processes. Based on these differences, it is likely that the coupling between degradation and secretion is both stress-dependent and cell-type specific. While chronic strain on autophagy via LRRK2 hyperactivity results in the release of exosomes and SLAM-EVs, we find that acute inhibition of autophagosome maturation in primary neurons via BafA1 prompts the release of a distinct vesicle population. Further, we find that BafA1 treatment of primary cortical mouse neurons induces the secretion of vesicles lacking CD63, contrasting with findings in other cell lines (65, 66). Thus, we observe cell-type specific responses to autophagy strain as well as differential responses to types of strain within a given cell type.

LRRK2 activity impacts many other cellular processes that could also impact secretion, particularly of exosomes (78, 79). Notably, many membrane- containing organelles are regulated and organized by Rab proteins, including effectors of LRRK2. For example, RAB10 can regulate exocytic cargo trafficking (80), and RAB7 impacts cargo sorting at the early endosome (81). LRRK2 can localize to the MVB (82), suggesting its activity could regulate MVB release or content. Thus, it is possible that multiple downstream effectors of LRRK2 impact exosome secretion. For example, LRRK2 can be activated at lysosomes via the conjugationof ATG8 to single membrane (CASM) pathway (83). However, our finding that LC3-II is fully membrane protected in SLAM-EVs suggests these vesicles are independent of CASM, and instead are generated by the incorporation of LC3-II into the double-membrane of the autophagosome, with the LC3-II on the inner leaflet of the inner autophagosomal membrane protease-resistant following secretion from the cell.

Our work has interesting implications regarding the role of secretion pathways in the context of neurodegenerative disease, suggesting that, at least in the short term, secretion can serve as an unburdening mechanism for neurons facing autophagic stress. Our findings complement recent work which found that increasing exosomal secretion via PIKFYVE inhibition was neuroprotective in several models of ALS (28). However, enhanced secretion of cellular debris, e.g., mitochondrial DNA, has the potential to induce a proinflammatory environment. Indeed, overall secretion is upregulated in models of neurodegenerative diseases including age-related macular degeneration (57) and Christianson Syndrome (84), and PD patients have higher levels of circulating mitochondria (85). Finally, upregulation of secretion could contribute to the spread of protein aggregates, as both alpha-synuclein (86) and amyloid-beta (87) can be released via secretory autophagy pathways. Thus, while enhanced secretion may have short-term benefits in staving off neuronal cell death, the described compensatory pathways may promote chronic neuroinflammation over longer timescales, contributing to age-dependent neurodegeneration.

Together, we posit that upregulated secretion is a compensatory mechanism that has immediate beneficial effects for neurons, but that over time, detrimental downstream impacts may contribute to disease progression.

## Materials and Methods

Detailed methods are available in the *SI Appendix, Materials and Methods*.

### EV Characterization.

Primary cortical neurons were isolated and cultured as previously described (16). iPSC lines were differentiated into cortical- like neurons and as previously described (16, 33). Details regarding ultracentrifugation parameters, density linear gradient subfractionation, proteinase K treatment, proteomic characterization, and smRNA characterization are listed in the *SI Appendix, Materials and Methods*.

### Microscopy.

Electron microscopy was performed by the University of Pennsylvania EM core. All live-cell imaging experiments were captured with a Perkin-Elmer Ultra VIEW Vox microscope fitted with a Visitron Orbital Ring-TIRF arm. All analysis was done blinded using FIJI.

## Supplementary Material

Appendix 01 (PDF)

Movie S1. Example video of control cortical neuron transfected with LC3mScarlet. Video captured at 2 Hz for three minutes. Example presumed fusion events noted with magenta circles. Events were counted if there was a stationary sudden burst of fluorescence that lasted between 10s–120s and no evidence of puncta trafficking out of frame. Scale bar = 5 μm.

Movie S2. Example video of LRRK2^G2019S^ cortical neuron transfected with LC3mScarlet. Video captured at 2 Hz for three minutes. Example presumed fusion events noted with magenta circles. Events were counted if there was a stationary sudden burst of fluorescence that lasted between 10s–120s and no evidence of puncta trafficking out of frame. Scale bar = 5 μm.

Movie S3. Example video of control neuron transfected with CD63pHluorin Video captured at 1frame every 5sec for 10 minutes (video trimmed from 20 minute video). Example exosome fusion events noted with cyan circles. Prolonged CD63 events noted with yellow circles. Scale bar = 5 μm.

Movie S4. Example video of hyperactive LRRK2 neuron transfected with CD63pHluorin Video captured at 1frame every 5sec for 10 minutes (video trimmed from 20 minute video). Example exosome fusion events noted with cyan circles. Prolonged CD63 events noted with yellow circles. Scale bar = 5 μm.

## Data Availability

The data, protocols, and key lab materials that were used and generated in this study are listed in a Key Resource Table, which will be deposited at Zenodo. The proteomics dataset has been deposited to MassIVE (MSV000095428) (88), and the transcriptomics dataset has been deposited to Sequence Read Archive (PRJNA1278743) (89). No code was generated for this study. Data cleaning, processing, analysis, and visualization were performed using GraphPad Prism and R. An earlier version of this manuscript was posted to bioRxiv (10.1101/2024.11.07.621551) (90).
