# Supplementary material for: Autophagic stress activates distinct compensatory secretory pathways in neurons
Source: Proc Natl Acad Sci U S A. 2025 Jul 7;122(28):e2421886122. doi: 10.1073/pnas.2421886122 (PMC12280970; doi:10.1073/pnas.2421886122)
Supplement: Supplementary file 1 — Appendix 01 (PDF) [file pnas.2421886122.sapp.pdf]

Supporting Information Text

## Supplemental Materials and Methods

### Experimental Lines

### Primary cortical neurons

All experiments used in this paper follow an approved protocol by the Institutional Animal Care and Use Committee at the University of Pennsylvania. Primary cortical neurons were isolated from either Lrrk2-p.G2019S KI mice (model #1390) (referred to as $LRRK2^{G2019S}$ ) or B6NTac mice (model #B6) (referred to as control), originally obtained from Taconic. For BafA1 experiments, C57BL/6J (model #000664) obtained from Jackson Laboratories were used. The isolation and culturing protocol followed is as previously published on protocols.io (https://doi.org/10.17504/protocols.io.81wgby723vpk/v1). Briefly, mouse cortices of both sexes were isolated from embryos at DIV 15.5, meninges were removed, and then cortices were digested with .35% Trypsin. Following digestion, cortices were triturated to single cells, counted, and then plated on imaging dishes (P35G- 1.5-20-C; MatTeK) that had been coated overnight with PLL (Sigma, # P1274). For initial plating, neurons were resuspended in attachment media containing MEM (ThermoFisher, # 11095-072) with 10% heat inactivated horse serum (ThermoFisher, # 16050-122), 33mM D-glucose (SigmaAldrich # G8769) and 1mM sodium pyruvate (Corning, # 36017004). Following 6 hours of incubation at 37°C, attachment media was replaced with maintenance media consisting of Neurobasal (ThermoFisher, # 21103-049), supplemented with 2% B-27 (Gibco, #17504-044), 33mM D-glucose, 2mM GlutaMAX (Gibco, #35050061), 100 U/mL penicillin and 100 mg/mL streptomycin (Gibco, #35050061.

For EV isolation, neurons were cultured until DIV11. To allow for the maximum number of collect EVs, no media was replaced, but, additional media was added at DIV7 to prevent dehydration and nutrient deprivation. For imaging experiments, neurons were imaged on DIV 7 following a 48-hour transfection using Lipofectamine 2000 (ThermoFisher, #11668019) and 1.5 µg total plasmid DNA. For ATG7 knock down experiments, siRNA (ATG7 or ctrl) was transfected in 48 hours prior to imaging. For SEC22B knock down experiments neurons were nucleofected (Lonza Amaxa Nucleofector II, program 05) with 3µg/million of shRNA (Dharmacon, SEC22B smart pool or scrambled control).

**Human iPSC derived neurons**

iPSCs (KOLF2.1J background WT and LRRK2-R1441H KI) were gifted to the Holzbaur lab from B. Skarnes as (Jackson Laboratories) through the iPSC Neurodegenerative Disease Initiative (iNDI). Both lines have a stably integrated doxycycline-inducible hNGN2 for neuronal differentiation and have been described previously(1) and characterized in further detail by our lab(2). iPSCs were cultured as previously described. Briefly, iPSCs were thawed in Essential 8 medium (ThermoFisher, #A151700) and plated onto Matrigel coated dishes and passaged twice before neuronal differentiation. Neurons were differentiated following an established protocol for $i^3$Neurons and Piggybac-delivered NGN2 neurons (https://www.protocols.io/view/ineuron-differentiation-from-human-ipscs-261ge348yl47/v1). After differentiation, neurons were cryopreserved in $i^3$Neuron media (BrainPhys Neuronal Medium (with 2% B-27 (Gibco, #17504-044), 10ng/mL BDNF (PeproTech 450-02), 10ng/mL NT-3 (PeproTech 450-03), and 1µg/mL Laminin (Corning, # 354232), with 10% DMSO, and 20% FBS). Cryopreserved differentiated KOLF2.1J neurons were thawed onto either 35-mm glass bottom dishes (300,000 neurons plated for live imaging experiments) or 10-cm tissue culture treated dishes (3 million neurons plated for EV isolation) coated with poly-L-ornithine overnight. Neurons were cultured for 21 days. ½ of $i^3$Neuron media was replaced every 3-4 weeks to prevent nutrient deprivation. For live imaging experiments, neurons were transfected using 3µg of DNA and Lipofectamine Stem (ThermoFisher) 2 days prior to imaging.

**Nanoparticle Tracking Analysis**

ZetaVIEW S/N 18-390 from Particle Metrix was calibrated using 100µM polycistronic beads. Extracellular vesicle samples isolated via the Qiagen ExoEasy (Qiagen, #76064) and were then diluted (1:1000 – 1:2000) in ddH2O directly before measurement to ensure accurate particle count. Samples were loaded onto ZetaVIEW from Particle Metrix and mode was set to size distribution with particle range set to 50nM- 1000nM. Minimum brightness was set to 20, sensitivity was set to 75 and shutter was set to 75. Prior to measurement, drift was confirmed to be minimal cell quality was confirmed to be “very good”. For each sample, the average particle count over 11 channels was taken. All experiments were performed at room temperature. Particles were counted using ZetaView (version 8.05.12 SP2) software, captured with a .712 mum/px camera. Original concentration was calculated based on starting dilution factor and

normalized to starting protein concentration. Particles count distribution for example plots were based on diluted counts taken directly from ZetaView software.

**CalceinAM – TIRF analysis**

Extracellular vesicles were pelleted via a 100X G spin for 90 minutes and resuspended in .2µM filtered 1X PBS. Diluted samples (1:1000) were then incubated with 10 µM Calcein AM (Invitrogen, C3099) at 37C for 30 minutes. 10 µL of solution was then applied to an individual PTFE Printed Slide well (Electron Microscopy Sciences, 63430-04) and incubated at room temperature for 10 minutes. Individual wells contain a bioadhesive surface which facilitates direct binding to slide. Wells were washed 3X with 10 µL of .2µM filtered 1X PBS before being mounted with for TIRF microscopy. For each sample, 6 individual planes were captured using Perkin-Elmer Ultra VIEW Vox fitted with an Orbital Ring-TIRF arm. Images were segmented using trainable 2D Weka Segmentation followed by object count in FIJI.

**EV isolation via ultracentrifugation**

For mass spectrometry analysis, 40mL of conditioned media was isolated from 10 million DIV11 primary cortical neurons (Lrrk2-p.G2019S KI or B6NTac genotype). Conditioned media was spun at 500g to remove dead cell and large debris. Supernatant was moved to thick-walled tubes fit for the Ti45 fixed angle rotor. All extracellular vesicles were pooled with a single 100,000g (RCF average) 90-minute spin at 4C, followed by a 1mL wash and subsequent 100,000g (RCF average) 90-minute spin in an Optima MAX XP ultracentrifuge fitted with a swinging bucket TLS 55 rotor. Pellet was resuspended in 100 µL of 1X PBS and 10% of each sample was set aside for protein quality analysis and concentration. Concentration was initially determined using a Qiagen Qubit kit and confirmed via Coomassie. 30µg of protein was collected for each sample and vacuum dried and stored at -80 prior to mass spectrometry analysis.

For immunoblotting, two populations of extracellular vesicles (LEVs and SEVs) were enriched via ultracentrifugation. 10-40 mL of cultured media isolated from 2-10 million primary cortical neurons was collected on DIV10. Media was spun at 500G for 10 minutes to remove cell debris. Supernatant was collected and then subjected to a 20,000g spin for 30 minutes using an Eppendorf 5417C centrifuge at 4°C. Pellets were washed with 1mL filtered 1X PBS before being

subjected to an additional 20,000g spin for 30 minutes at 4C. Washed pellet was resuspended in 75 µL of 1X PBS before being denatured for immunoblotting (Large EVs/P20). Supernatant was collected and then spun at 100,000g (RCF average) for 90-minutes at 4°C using an Optima XPN 80 ultracentrifuge fitted with a swinging bucket SW41 TI rotor. Following initial spin, supernatant was removed and pellet was resuspended in 1X PBS and then subjected to an additional 100,000X G spin using an Optima MAX XP ultracentrifuge fitted with a swinging bucket TLS 55 rotor for 90 minutes at 4°C. Small EVs/P100 pellet was collected following second spin and resuspended in 75 µL of 1X PBS before being denatured for immunoblotting.

For the Proteinase K protection assay, isolated EV samples (P20 or P100) were equally divided into three tubes to be incubated with 1) 1X PBS, 2) 5 µg/ml proteinase K, or 3) 5 µg/ml proteinase K + 2% Triton X for 15 min on ice. To stop enzyme activity, PMSF (100 mM) was added for 5 minutes before samples were denatured for immunoblotting.

**Density linear gradient subfractionation**

EV populations were first isolated as described above. Isolated pellets (Small EVs/P100 or Large EVs/P20) from 40mL of conditioned media of 10 million DIV12 LRRK2$^{G2019S}$ neurons were then resuspended in 50% iodixanol. The EV/iodixanol mixture was loaded into the bottom of a 13.2 mL open-top thinwall ultraclear tube (Beckman Coulter #344059). A linear gradient of 45% iodixanol-1XPBS was then loaded on top of the EV fraction. A control tube was also loaded with a linear gradient that lacked any EV sample to measure the density of each fraction. The linear gradients were then subjected to 180,000xg spin for 18 hours. 12 fractions were isolated by puncturing the thinwall tube and extracting with a syringe. Each of the 12 samples was concentrated by dialysis (Repligen, #131489) overnight in .1XPBS. Following dialysis, fractions were dried via vacuum centrifugation, and then resuspended in denaturing buffer for immunoblot analysis.

**Protein extraction and digestion for proteomics**

EV pellets isolated via 100,000 x g spin were solubilized in extraction buffer (5% sodium dodecyl sulfate (Affymetrix), 50mM TEAB (pH 8.5, Sigma), and protease inhibitor cocktail (Roche cOmplete, EDTA free)). Samples were sonicated and then centrifuged at 3000g for 10 minutes before protein concentration was measured by intrinsic tryptophan fluorescence. 10ug of

each sample was digested per the S-Trap Micro (Protifi) manufacturer's protocol(3). After digestion, peptides were eluted and organic solvent was dried off via vacuum centrifugation and reconstituted in 0.1% TFA containing iRT peptides (Biognosys, Schlieren, Switzerland). Peptide concentration was measured at OD280 and samples were adjusted to 400 ng/ul.

**Mass Spectrometry data acquisition**

Following sample preparation, samples were randomized and analyzed on an Exploris 480 mass spectrometer (Thermofisher Scientific San Jose, CA) coupled with an Ultimate 3000 nano UPLC system and an EasySpray source. 5ul of sample was loaded onto an Acclaim PepMap 100 75um x 2cm trap column (Thermo) at 5uL/min, and separated by reverse phase (RP)-HPLC on a nanocapillary column (75 μm id × 50cm 2um PepMap RSLC C18 column (Thermo)). Mobile phase A consisted of 0.1% formic acid and mobile phase B of 0.1% formic acid/acetonitrile. Peptides were eluted into the mass spectrometer at 300 nL/min with each RP-LC run comprising a 105-minute gradient from 3% B to 45% B.

The following mass spectrometer settings for data independent acquisition (DIA) were used: First, a full MS scan at 120,000 resolution, with a scan range of 350-1200 m/z and normalized automatic gain control (AGC) target of 300%, and maximum inject time. This was followed by variable (DIA) isolation windows, MS2 scans at 30,000 resolution, a normalized AGC target of 1000%, and automatic injection time. The default charge state was 3, the first mass was fixed at 250 m/z, and the normalized collision energy for each window was set at 27.

**Proteomic bioinformatics analysis**

Raw data were searched using Spectronaut(4, 5) and proteomics data processing and statistical analysis were conducted in R. The MS2 intensity values generated by Spectronaut were utilized for analyzing the entire proteome dataset. Following log2 transformation and normalization the median value for each sample was subtracted to produce an expression value. Only proteins with complete values in at least one cohort were included. A Limma t-test was used to identify proteins with differential abundance. Lists of differentially abundant proteins were generated based on criteria of adjusted P.Value $<0.05$.

For ontology analysis, PANTHER was used and terms were compared to available MitoCarta 3.0(6), SynGo(7), and EV datasets(8).

**Small RNA Isolation and Sequencing**

To isolate small RNAs, the miRNeasy kit (Qiagen) was used. Following isolation, RNA sample quality was assessed by High Sensitivity RNA Tapestation (Agilent Technologies Inc.) and quantity was determined via Qubit 2.0 RNA HS assay (ThermoFisher). Library construction is performed based on manufacturer's recommendation for the SMARTer smRNA library preparation kit (Takara Bio USA Inc). Final library quantity was measured by KAPA SYBR® FAST qPCR and library quality evaluated by TapeStation D1000 ScreenTape (Agilent Technologies). Equimolar pooling of libraries was performed based on QC values and sequenced on an Illumina NovaSeq S4 (Illumina, California, USA) with a read length configuration of 150 PE for 20M PE reads per sample (20M in each direction).

**miRNA Differential Expression Analysis**

Raw data reads were filtered to remove low quality reads or redundant reads. Reads were further filtered to ensure N content was greater than 10% reads, match greater than or equal to 15bp and mismatch number less than or equal to 3bp. Fastp was used for data quality filtering to remove adapter sequences from read.

For miRNA identification and quantification, we used COMPSRA, a comprehensive platform for small RNA-Seq data analysis. To align the clean reads to the reference genome, COMPSRA uses STAR as its default RNA sequence aligner with default parameters. The aligned reads are quantified and annotated. DESeq2 software was used to analyze the DEG for samples with biological replicates and edgeR was used for the samples without replicates. During the analysis, samples should be firstly grouped so that comparison between every two groups as a control-treatment pairwise can be done later. During the process, Fold Change$\geq$2.00 and padj$\leq$0.05 are set as screening criteria. Fold Change (FC) indicates the ratio of expression levels between two samples (groups).

**Immunoblotting**

For whole cell lysate samples, DIV11 cultured neurons were lysed in RIPA buffer (50 mM Tris-HCl supplemented with150 mM NaCl, 0.1% Triton X-100, 0.5% sodium deoxycholate and 0.1% SDS, pH=7.4, 1X Halt Protease and phosphatase inhibitor) at 4C for 30 minutes before being centrifuged at 13,000 RPM for 10 minutes. Concentration of all samples was confirmed via Pierce BCA Protein Assay Kit (ThermoFisher #23225) and then denatured in 1X denaturing buffer containing SDS and boiled at 95C for 10 minutes. For all EV samples, EVs were enriched via ultracentrifugation, and equal volume was taken from each replicate before being denatured in 1X denaturing buffer and boiled at 95C for 10 minutes. All EV protein samples were loaded onto gradient SDS-PAGE gels (BioRad #4568084) while lysates and plasma were loaded onto to fixed percent SDS-PAGE gel to be resolved. Following protein separation, resolved proteins were transferred to PVDF membranes (#), and dried overnight. Total protein was determined using Revert™ 700 Total Protein Stain (Licor #926-11021) following the manufacturer's protocol. Membranes were destained and then blocked using EverBlot Blocking Buffer (Bio-Rad #) before being incubated with primary antibodies over night at 4C (Antibodies listed in primary resource table). Following 3X washes with 1X TBST, membranes were incubated with appropriate secondary antibodies and band intensities were quantified using Image Studio™ Software (Li-COR).

**Electron Microscopy**

Isolated Large EVs/P20 from control and RRK2 neurons were resuspended in EM fixation buffer (2.5% glutaraldehyde, 2.0% paraformaldehyde in 0.1M sodium cacodylate buffer, pH 7.4) overnight at 4°C. Fixed samples were then transferred to the Electron Microscopy Resource Laboratory at the University of Pennsylvania. Fixed Large EVs/P20 samples were then postfixed in 2.0% osmium tetroxide and washed. Samples were dehydrated through a graded ethanol series, after which the tissue was infiltrated and embedded in EMbed-812 (Electron Microscopy Sciences, Fort Washington, PA). Thin sections were stained with lead citrate and examined with a JEOL 1010 electron microscope fitted with a Hamamatsu digital camera and AMT Advantage image capture software. Regions with high numbers of ~1micron vesicles were imaged.

**ExoView R100**

Tetraspanin CD9, CD63 and CD81 distribution were analyzed using the ExoViewR100 platform using the Leprechaun mouse tetraspanin ExoView kits (Unchained Labs # 251-1046) following the kit assay protocol. Isolated EVs (P100) were diluted 1:100 in the proprietary incubation solution II. 50 µL of each sample was placed inside a Falcon 24-well cell culture plate, flat bottom (Fisher Scientific Catalogue number 08-772-1) for the capture of EV hamster anti mouse CD81antigen (Clone Eat-2) as well as controls Armenian hamster isotype IgG (Clone HTK888) and rat isotype IgG2ak (Clone RTK2758). Samples were incubated for 16 h at RT and then washed 3X with Solution A on an ELISA microplate orbital shaker at 500 rpm (Fisherbrand™ Fisher Scientific # 88-861-023). Chips were then incubated with an antibody cocktail made of 0.6 µL Amrmenia hamster anti mouse CD81$^{+(Clone}$ Eat-2) conjugated with Alexfluor 555, 0.6 µL rat anti mouse CD63(Clone NVG-2) conjugated with Alexfluor 647, and 0.6 µL of rat anti mouse CD9 (Clone MZ3) conjugated with Alexfluor 488 in 300 µL of blocking solution for 1h at RT in on orbital shaker at 500 rpm. Chips were washed with 1X Solution A, followed by 3X washes with 1X Solution B and at 500 rpm. Image acquisition from each chip was carried out using the ExoView® R100 platform, and the data were analyzed by the ExoView Analyzer software version 3.2 (NanoView Biosciences). The images of the acquisition were visually inspected and all the artifacts onto the spots were manually removed from the analysis. Non-specific binding was checked on the mouse isotype control IgG spots. The cut off was manually established for all the chip to exclude the majority of the signal (> 90%) captured on the isotype control.

**TIRF microscopy**

To assay fusion events of CD63-pHluorin, CD9-pHluorin, or LC3, primary cortical neurons were transfected 48 hours prior to imaging. If neurons were treated with MLI-2, drug was applied in maintenance media 1 hour prior to imaging. Immediately before imaging, neuronal maintenance media was replaced with Hibernate E supplemented with 2% B-27 and 22mM D glucose. All live experiments were captured with a Perkin-Elmer Ultra VIEW Vox microscope fitted with a Visitron Orbital Ring-TIRF arm. A CFI Apo TIRF 100X (1.49 NA) oil immersion objective was used for all experiments and videos were captured using VisiView (Visitron). For CD63pHluorin secretion events, cells were imaged over a 5-minute time frame at 2Hz with perfect focus. To assess the longer-term CD63phluorin events, cells were imaged over a 20-minute time frame

(1frame/5seconds) with perfect focus. For LC3 experiments, cells were imaged over a 5-minute time frame at 2Hz.

All analysis was done blinded by two independent experimenters. Videos were aligned in FIJI using the Fast4DReg plugin. The first frame was then subtracted from all subsequent frames using the Image Calculator in FIJI. Fusion events were defined as a rapid increase in GFP signal that persisted for 5 or more frames. Cells were analyzed if they remained in focus for 75% of the imaging time minutes and appeared healthy throughout.

**Plasma isolation**

Following IACUC approved euthanasia and decapitation, 500µL of trunk blood from 1yo Lrrk2-p.G2019S KI mice (model #1390) or B6NTac mice (model #B6) was collected into EDTA collection tubes (Sarstedt Inc #NC9990563). Blood was then spun at 2,000g for 10 minutes. 100µL of supernatant (plasma) was carefully collected into a new Eppendorf tube, diluted with 200µL of 1XPBS, and then denatured (final concentration 1X denaturing buffer with SDS, 20 minutes at 95°C) for immunoblotting.

**Cell Death Assay**

Primary cortical DIV7 neurons were treated with varying concentrations of either GW4869 (Tocris #6741) or Y27632 (Tocris #1254) for two hours. A single drop of CellEvent Caspase-3/7 Green Detection Reagent (ThermoFisher R37111) was added to individual dishes and incubated at 37 °C, 5% CO2 for 30 min. Neurons were treated with Hoechst nuclear stain 10 minutes prior to imaging. Cells were imaged using Leica DMI6000B inverted epifluorescence microscope (20X) equipped a climate-controlled chamber. Analysis was performed using CellPose to quantify the total number of cell bodies in Hoechst and the number of Caspase3/7 positive cells.

**Statistics**

Statistical tests of all NTA, immunoblotting, fixed, and live imaging experiments were performed in Graphpad Prism V10. For NTA analysis, the concentration of detected particles was used to determine total number of secreted particles. Total number of secreted particles were compared using a two-tailed t-test. For immunoblot analyses in which the comparison was between two

groups, a Kruskal-Wallis test was used. For immunoblot analyses comparing three or more groups, a two-way ANOVA with Šídák's multiple comparisons test was used. For live-cell and fixed imaging, an unpaired t-test was performed on the mean of the biological replicates to determine significance. Biological replicates (n) are displayed as larger data points in superplot graphs with technical replicates as smaller, transparent points. In all cases, significance was defined as a p-value $<0.05$ and the detected p-value was displayed in each figure. Statistics used for proteomic and transcriptomic results are specified in their respective methods sections.

**Figure S1**

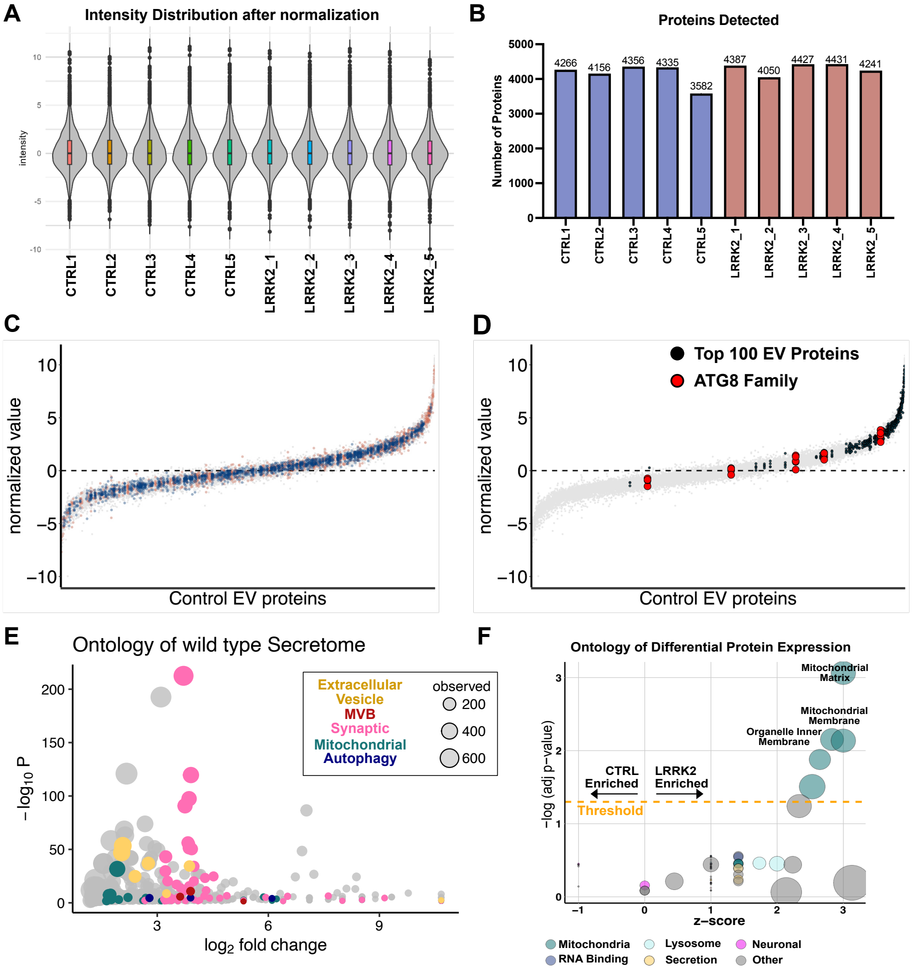


**A)** Intensity distribution of detected proteins for all samples following normalization. Intensity of 0 represents median abundance.

**B)** Total number of proteins detected across samples used in quantitative proteomic analysis.

**C)** Proteins detected in EVs isolated from 5 samples of control neurons ranked by abundance. Median protein abundance = 0. Large EV proteins blue, Small EV proteins orange.

**D)** Proteins detected in control secretome ranked by abundance. Black dots indicate proteins defined as top 100 EV-associated cargo. Red dots indicate members of ATG 8 family.

**E)** Bubble plot representation of ontology terms of top 50% of detected proteins in wild type secretome. Ontology analysis of GO cellular component by PANTHER. Each bubble depicts unique GO term and size of bubble represents number of proteins within term that was detected. Bubbles are pseudo coated by terms indicated in box inset.

**F)** Bubble plot representation gene ontology terms from proteins differentially expressed between control and LRRK2$^{G2019S}$ EVs. Significance threshold determined by p-value of differential abundance analysis. Each bubble depicts unique GO term. Bubbles are pseudo colored based on broader terms indicated below. Size of bubble represents number of proteins representing that term.

## Figure S2

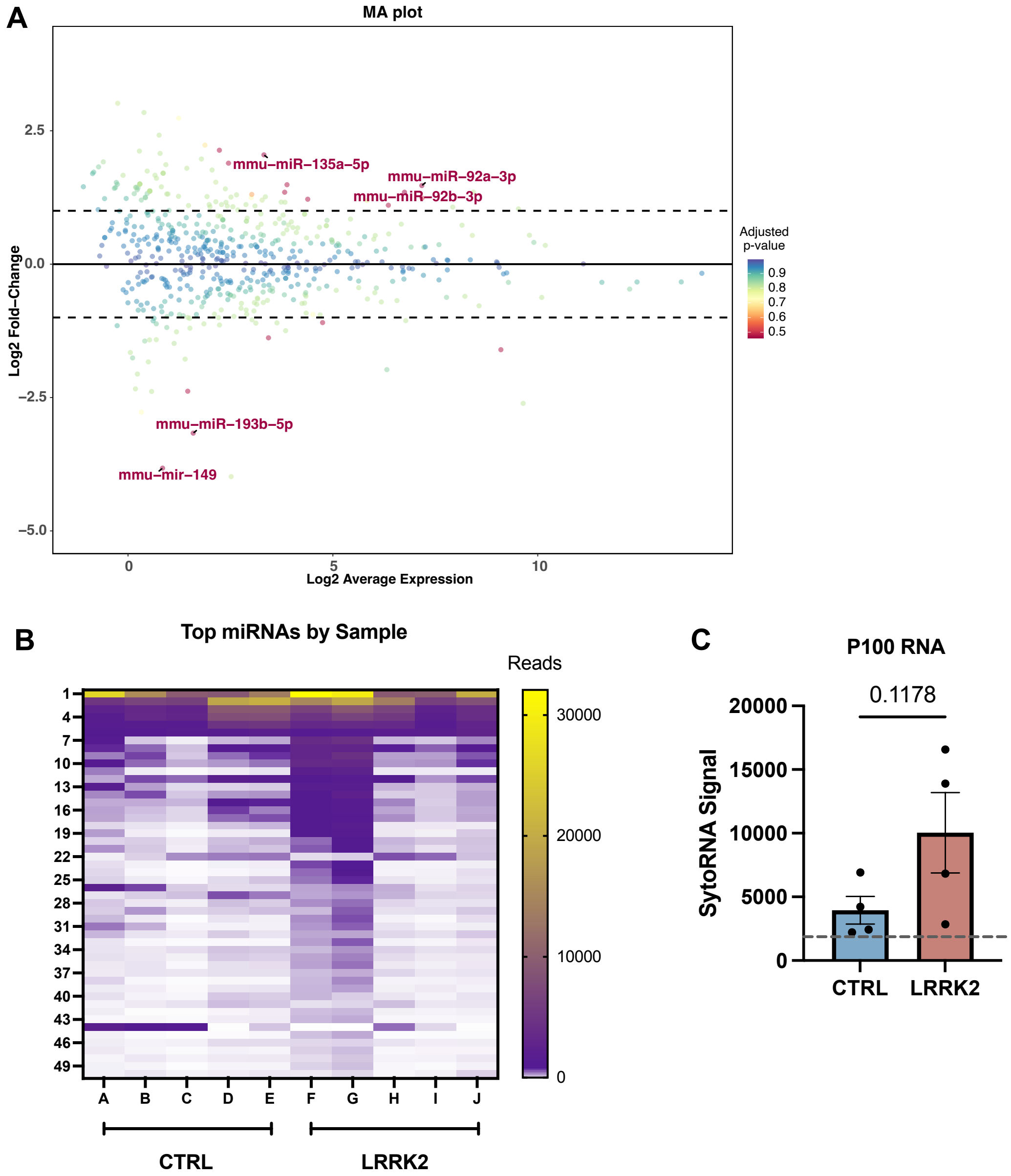


**A)** Heatmap of number of reads per individual sample of the 50 miRNAs detected in control (Samples A-E) and LRRK2$^{G2019S}$ (Samples F-J).

**B)** Differential expression of miRNAs from LRRK2$^{G2019S}$ and wild type secreted transcriptomes. No miRNAs significantly different between genotypes.

**C)** Fluorescence intensity of SYTO ® RNASelect™ Green Fluorescent Cell Stain measured by fluorimeter of P100 fractions isolated from control and LRRK2$^{G2019S}$ primary cortical neurons.

## Figure S3

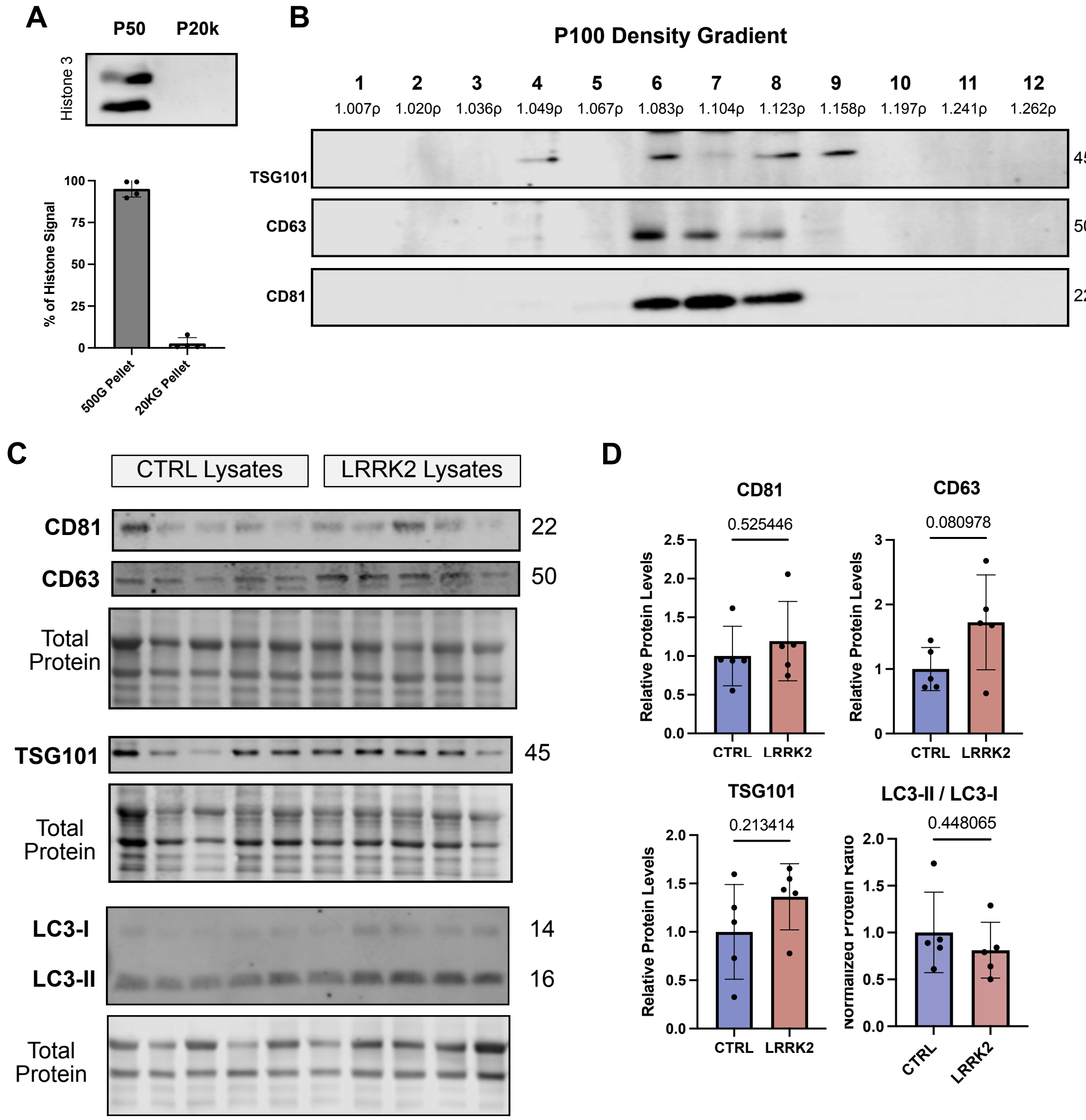


**A)** Representative western blot of Histone3 signal isolated from 500xg pellet and corresponding 20,000xg pellet (left) and quantification of relative Histone3 signal in 500xg pellet vs. 20,000xg pellet (right).

**B)** Representative immunoblot of isolated fractions from P100 vesicles subfractionated by buoyant linear density gradient.

**C)** Representative western blots of isolated cell lysates from wild type and LRRK2$^{G2019S}$ murine cortical neurons. Detected protein and corresponding molecular weight indicated.

**D)** Quantifications of relative levels of detected band intensities for CD81, CD63, TSG101, and the ratio of LC3-II/LC-1 from cell lysates of control (blue) and LRRK2$^{G2019S}$ (red) neurons. Individual replicates represented by black dots. N = 5, two-tailed t-test comparing biological replicates, error bars represent SEM.

**Figure S4**

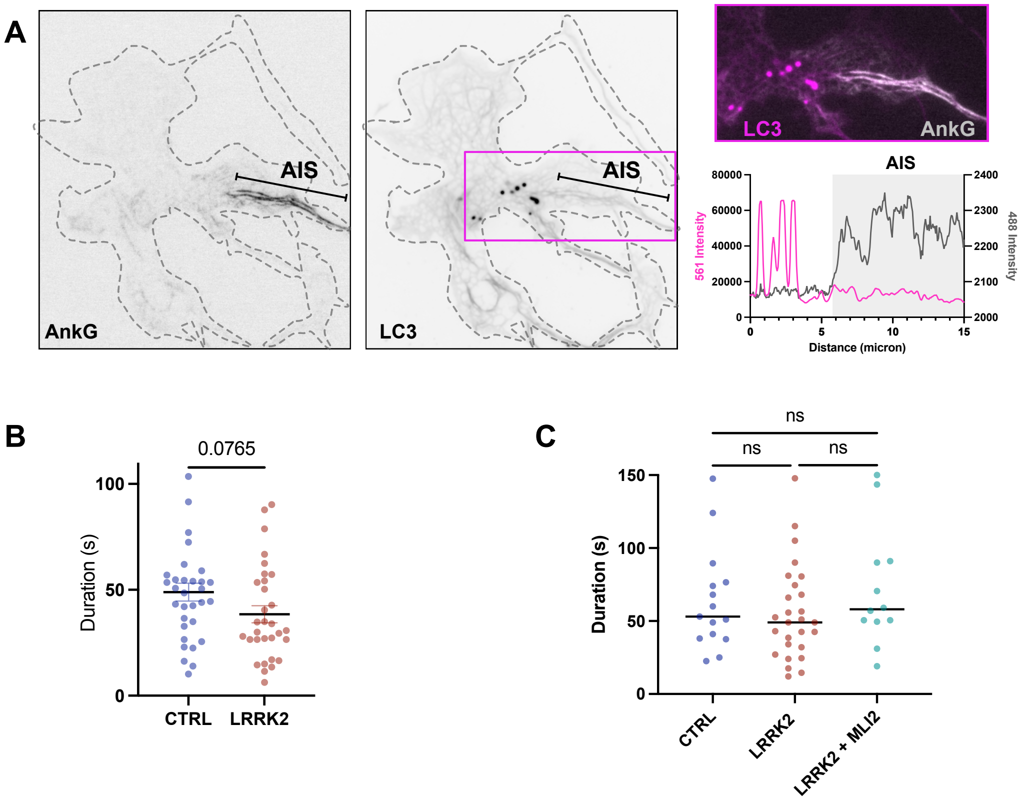


**A)** Representative image LRRK2$^{G2019S}$ neuron transfected with AnkyrinG and LC3mScarlet. Axon Initiation Segment (AIS) indicated by bar. Insets of AIS and neighboring somatodendritic compartment. Representative linescan of LC3mScarlet (magenta) and AnkyrinG (grey) of axon and neighboring somatodendritic compartment.

**B)** Representative western blot of cell lysate isolated from LRRK2$^{G2019S}$ neurons following 72-hours after electroporation with siRNA against scrambled control or ATG7.

**C)** Quantification of duration of LC3mScarlet fluorescent events in control and LRRK2$^{G2019S}$ neurons.

**D)** Quantification of duration of CD63pHluorin secretion events in control and LRRK2$^{G2019S}$ neurons.

Figure S5

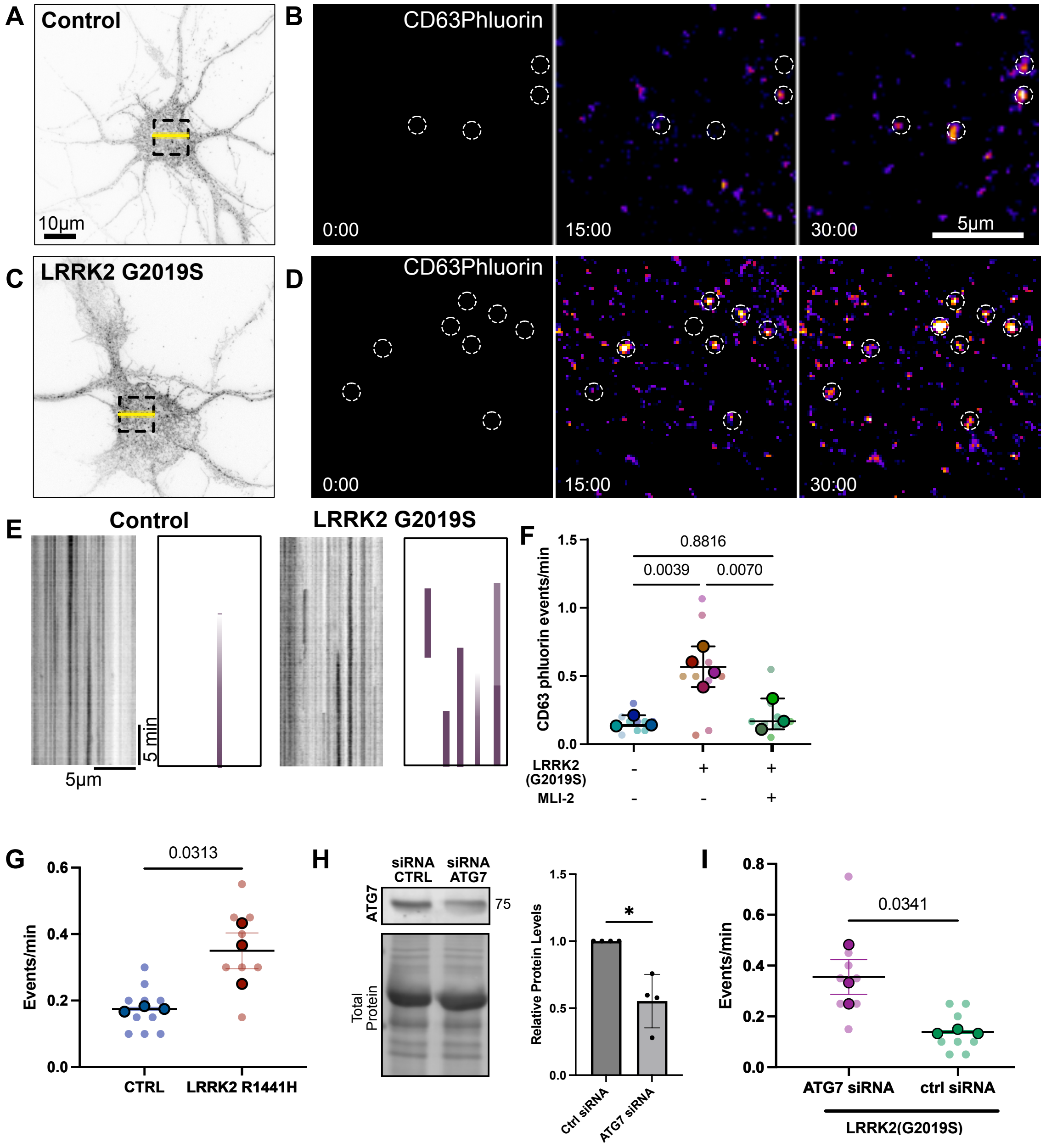

A
Control
10μm
B
CD63Phluorin
0:00
15:00
30:00
5μm
C
LRRK2 G2019S
D
CD63Phluorin
0:00
15:00
30:00
E
Control
LRRK2 G2019S
5 min
5μm
F
CD63 phluorin events/min
0.8816
0.0039
0.0070
LRRK2 (G2019S)
MLI-2
G
Events/min
0.0313
CTRL
LRRK2 R1441H
H
siRNA CTRL
siRNA ATG7
ATG7
75
Total Protein
Relative Protein Levels
*
Ctrl siRNA
ATG7 siRNA
I
Events/min
0.0341
ATG7 siRNA
ctrl siRNA
LRRK2(G2019S)

**A)** Representative image of CD63pHluorin expressing control neuron. Dashed box indicates panels depicted in panel B. Yellow line indicates kymograph depicted in E.

**B)** Representative time series of persisting CD63pHluorin fluorescent events (>3min) in control neuron. The first frame was subtracted from all subsequent frames. Time stamp indicated in each frame. Dashed circles indicate fusion events.

**C)** Representative image of CD63pHluorin expressing LRRK2$^{G2019S}$ neuron. Dashed box indicates panels depicted in panel D. Yellow line indicates kymograph depicted in panel E.

**D)** Representative time series of persisting CD63pHluorin fluorescent events (>3min) in LRRK2$^{G2019S}$ neuron. first frame was subtracted from all subsequent frames. Time stamp indicated in each frame. Dashed circles indicate fusion events.

**E)** Representative kymographs and schematic indicating persistent CD63pHluorin fluorescent events over time in control and LRRK2$^{G2019S}$ neurons.

**F)** Quantification of persistent CD63pHluorin events in control, LRRK2$^{G2019S}$, and LRRK2$^{G2019S}$ + MLI-2 primary cortical neurons. Superplot indicating biological and technical replicates. N=4, ordinary one-way ANOVA with Tukey's multiple comparison test of biological replicates, error bars represent SEM.

**G)** Quantification of CD63pHluorin secretion events in control, LRRK2$^{R1441H}$ KOLF2.1J neurons. Superplot indicating biological and technical replicates. N=3, two-tailed t-test comparing biological replicates, error bars represent SEM.

**H)** Representative blot and quantification of relative amounts of ATG7 following knockdown with ATG7 siRNA

**I)** Quantification of CD63pHluorin events in LRRK2$^{G2019S}$ cortical neurons transfected with either scrambled CTRL siRNA or ATG7 siRNA. Superplot indicating biological and technical replicates. N=3, two-tailed t-test comparing biological replicates, error bars represent SEM.

**Figure S6**

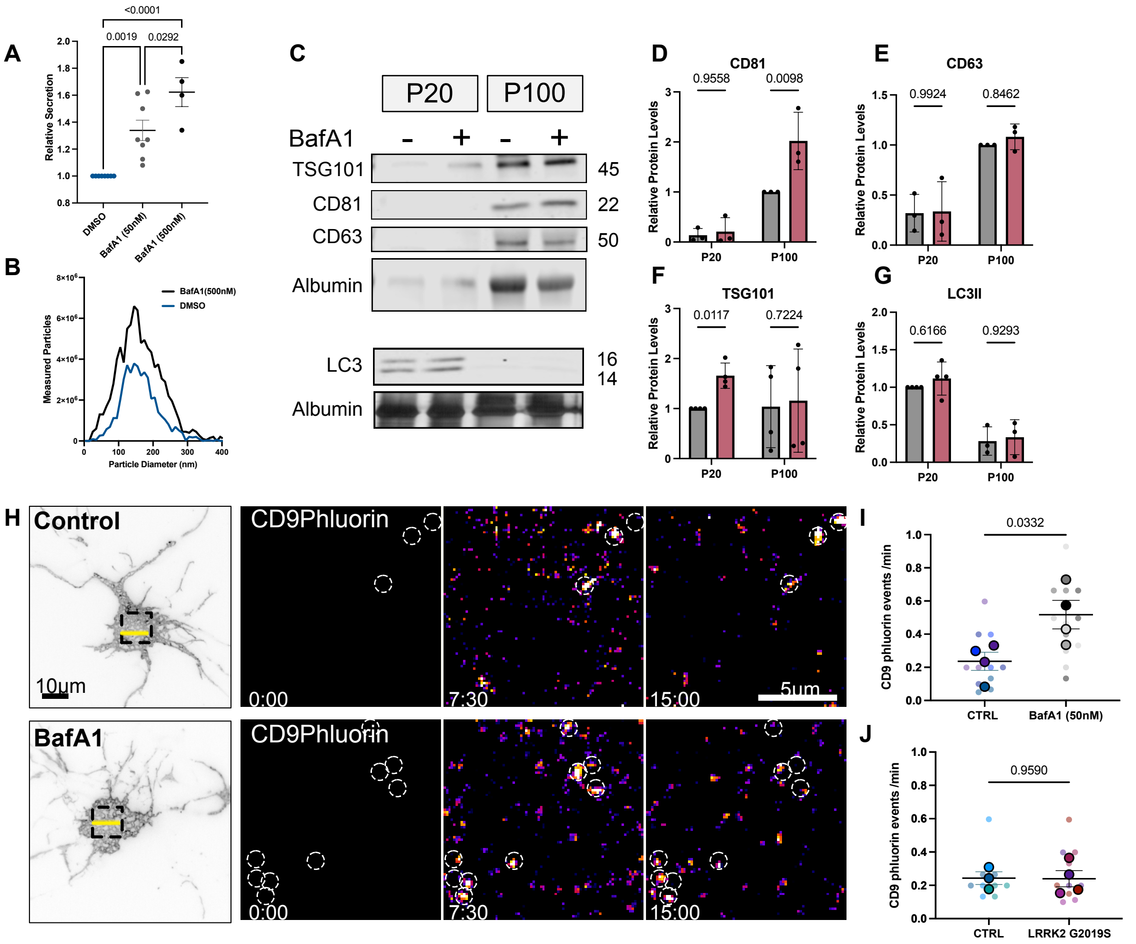


**A)** Quantification of Nanoparticle Tracking Analysis (NTA) of secreted particles isolated from DIV11 control primary cortical neurons treated with BafA1 for 2 hours. Ordinary one-way ANOVA with Tukey's multiple comparison test, error bars represent SEM.

**B)** Representative size distribution of measured particles released from control primary cortical neurons treated with DMSO or 500nM BafA1 for 2 hours.

**C)** Representative western blots of isolated cell lysates from primary cortical control neurons treated with DMSO control or 500nM BafA1 for 2 hours.

**D-G)** Quantifications of relative levels of detected band intensities for **D)** CD81, **E)** CD63, **F)** TSG101, and **G)** LC3II in DMSO (grey) vs. BafA1 (blue) treated control neurons. N = 4. 2way ANOVA with Šídák's multiple comparison test. Error bars indicate SEM.

**H)** Representative image of CD9pHluorin expressing control neuron treated with DMSO (top) or 50nM BafA1 (Bottom). Dashed box indicates zoomed in time series. First frame subtracted from subsequent frames. Dashed circles indicate fusion events.

**I)** Quantification of CD9pHluorin events in control neurons treated with either DMSO (CTRL) or 50nM BafA1. N=4, two-tailed t-test, error bars represent SEM.

**J)** Quantification of CD9pHluorin events in control and LRRK2$^{G2019S}$ neurons. N=4, two-tailed t-test comparing biological replicates, error bars represent SEM.

**Figure S7**

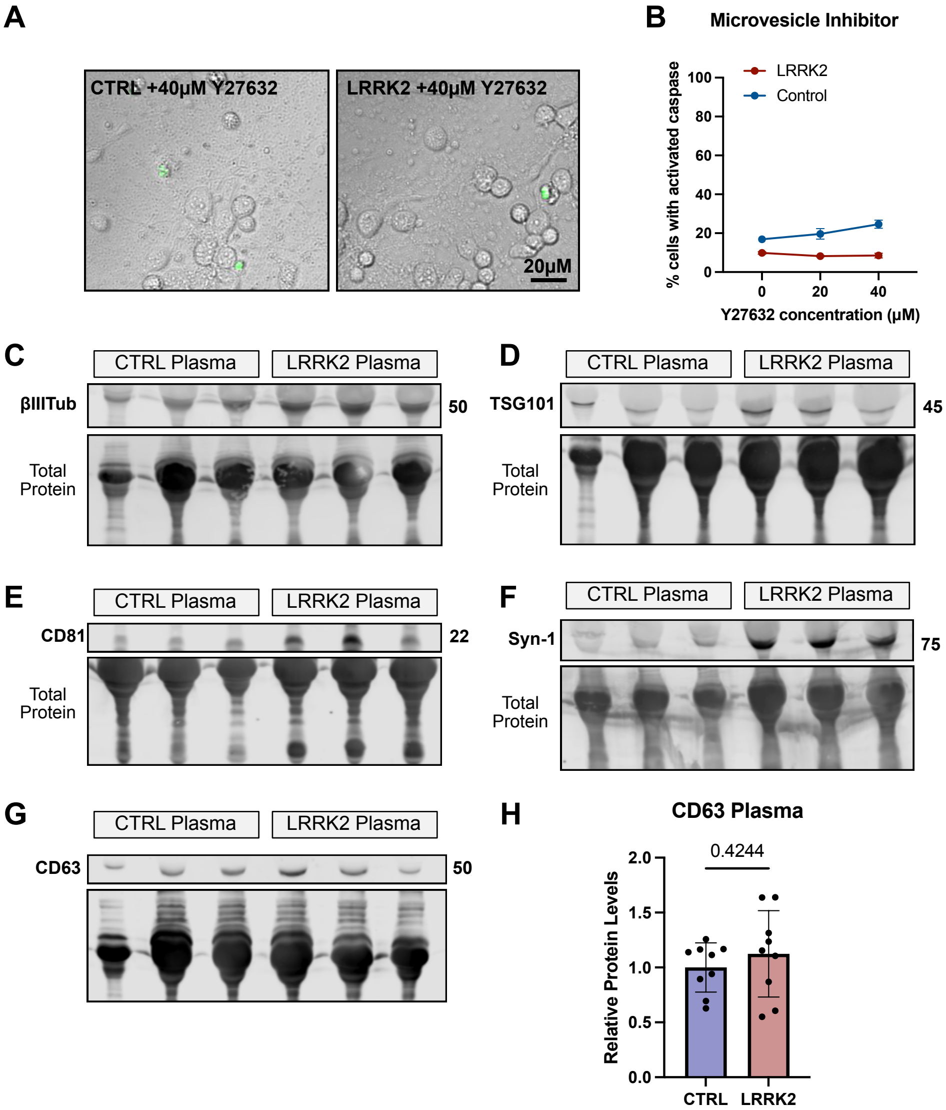


**A)** Example images of control and LRRK2$^{G2019S}$ neurons treated with 40 µM of the microvesicle inhibitor, Y27632. DIC images with overlay of activated CellEvent Caspase3/7 ready probe which is an early indicator of apoptosis. Scale bar = 20µM

**B)** Quantification of percent of neurons with activated Caspase 3/7 following two hours of treatment with varying levels of Y27632. N=3, 2way ANOVA with Šídák's multiple comparison test. Error bars indicate SEM.

**C-G)** Representative western blots of isolated plasma from one year old control and LRRK2$^{G2019S}$ mice against **C)** Beta III Tubulin, **D)** TSG101 **E)** CD81F) Synapsin-1 and **G)** CD63.

**H)** Quantification of relative band intensity of isolated plasma from one year old control and LRRK2$^{G2019S}$ mice for CD63. N=11, two-tailed t-test, error bars represent SEM.

**Supplemental Video 1**
Example video of control cortical neuron transfected with LC3mScarlet. Video captured at 2 Hz for three minutes. Example presumed fusion events noted with magenta circles. Events were counted if there was a stationary sudden burst of fluorescence that lasted between 10s-120s and no evidence of puncta trafficking out of frame. Scale bar = 5 µm.

**Supplemental Video 2**
Example video of LRRK2$^{G2019S}$ cortical neuron transfected with LC3mScarlet. Video captured at 2 Hz for three minutes. Example presumed fusion events noted with magenta circles. Events were counted if there was a stationary sudden burst of fluorescence that lasted between 10s-120s and no evidence of puncta trafficking out of frame. Scale bar = 5 µm.

**Supplemental Video 3**
Example video of control neuron transfected with CD63pHluorin Video captured at 1frame every 5sec for 10 minutes (video trimmed from 20 minute video). Example exosome fusion events noted with cyan circles. Prolonged CD63 events noted with yellow circles. Scale bar = 5 µm.

**Supplemental Video 4**
Example video of hyperactive LRRK2 neuron transfected with CD63pHluorin Video captured at 1frame every 5sec for 10 minutes (video trimmed from 20 minute video). Example exosome fusion events noted with cyan circles. Prolonged CD63 events noted with yellow circles. Scale bar = 5 µm.

**Supplemental References:**


1. C. B. Pantazis, *et al.*, A reference human induced pluripotent stem cell line for large-scale collaborative studies. *Cell Stem Cell* **29**, 1685-1702.e22 (2022).

2. D. Dou, E. M. Smith, C. S. Evans, C. A. Boecker, E. L. F. Holzbaur, Regulatory imbalance between LRRK2 kinase, PPM1H phosphatase, and ARF6 GTPase disrupts the axonal transport of autophagosomes. *Cell Reports* **42**, 112448 (2023).

3. A. Zougman, P. J. Selby, R. E. Banks, Suspension trapping (STrap) sample preparation method for bottom-up proteomics analysis. *Proteomics* **14**, 1006–1000 (2014).

4. The MaxQuant computational platform for mass spectrometry-based shotgun proteomics | Nature Protocols. Available at: https://www.nature.com/articles/nprot.2016.136 [Accessed 21 October 2024].

5. R. Bruderer, *et al.*, Extending the limits of quantitative proteome profiling with data-independent acquisition and application to acetaminophen-treated three-dimensional liver microtissues. *Mol Cell Proteomics* **14**, 1400–1410 (2015).

6. S. Rath, *et al.*, MitoCarta3.0: an updated mitochondrial proteome now with sub-organelle localization and pathway annotations. *Nucleic Acids Res* **49**, D1541–D1547 (2021).

7. F. Koopmans, *et al.*, SynGO: An Evidence-Based, Expert-Curated Knowledge Base for the Synapse. *Neuron* **103**, 217-234.e4 (2019).

8. S. V. Chitti, *et al.*, Vesiclepedia 2024: an extracellular vesicles and extracellular particles repository. *Nucleic Acids Res* **52**, D1694–D1698 (2024).
